# Dataset of milk whey proteins of two indigenous greek goat breeds

**DOI:** 10.1016/j.dib.2016.06.038

**Published:** 2016-06-28

**Authors:** Athanasios K. Anagnostopoulos, Angeliki I. Katsafadou, Vasileios Pierros, Evangelos Kontopodis, George C. Fthenakis, George Arsenos, Spyridon Ch. Karkabounas, Athina Tzora, Ioannis Skoufos, George Th. Tsangaris

**Affiliations:** a Proteomics Research Unit, Center of Basic Research II, Biomedical Research Foundation of the Academy of Athens, Athens, Greece; b Veterinary Faculty, University of Thessaly, Karditsa, Greece; c Laboratory of Animal Husbandry, School of Veterinary Medicine, Aristotle University of Thessaloniki, Thessaloniki, Greece; d Cell and Molecular Physiology Unit, Laboratory of Experimental Physiology, Medical School, University of Ioannina, Ioannina, Greece; e Department of Animal Production, Technological Educational Institute of Epirus, Arta, Greece

**Keywords:** Foodomics, milk whey, Capra prisca breed, Skopelos Breed, LC-MS/MS, Greek goat

## Abstract

Due to its rarity and unique biological traits, as well as its growing financial value, milk of dairy Greek small ruminants is continuously attracting interest from both the scientific community and industry. For the construction of the present dataset, cutting-edge proteomics methodologies were employed, in order to investigate and characterize, for the first time, the milk whey proteome from the two indigenous Greek goat breeds, *Capra prisca* and Skopelos. In total 822 protein groups were identified in milk whey of the two breeds, The present data are further discussed in the research article “Milk of Greek sheep and goat breeds; characterization by means of proteomics” [1].

**Specifications Table**

**Table:** 

Subject area	*Foodomics, Veterinary science*
More specific subject area	*Capra prisca and Skopelos goat milk proteome*
Type of data	*Excel file, Figures*
How data was acquired	1D-nanoLC-MS/MS, bottom-up proteomics. Dionex Ultimate 3000 nanoHPLC system coupled to an LTQ Velos *Orbitrap Elite mass spectrometer* (Thermo Scientific, Rockford, IL, USA). PepMap® RSLC, C18, 100 Å, 3 μm-bead-packed 15 cm column and 2 μm-bead-packed 50 cm column (Thermo Scientific). Proteome Discoverer 1.4 software (Thermo Scientific), Sequest search engine searching the *Rumintae* *.fasta databases for milk of goats.
Data format	*Analyzed*
Experimental factors	*Milk samples from the indigenous Greek goat breeds Capra prisca and Skopelos, were systematically collected and analyzed in order to characterize the protein content of the milk of each breed.*
Experimental features	*Whole proteome analysis of milk whey*
Data source location	*Athens, Greece*
Data accessibility	*Datasets are directly provided with this article*

**Value of the data**

• The proteome dataset of milk whey from two indigenous Greek goat breeds was reported for the first time.
• The data can be used to govern future steps in optimizing characteristics and features of goat milk products.
• The comparative analysis of the data could lead to new dairy products with specific nutritional characteristics for the human health.
• The present data can be used for traceability purposes of dairy products from these goat breeds.

## 1 Data

**Fig. 1:**
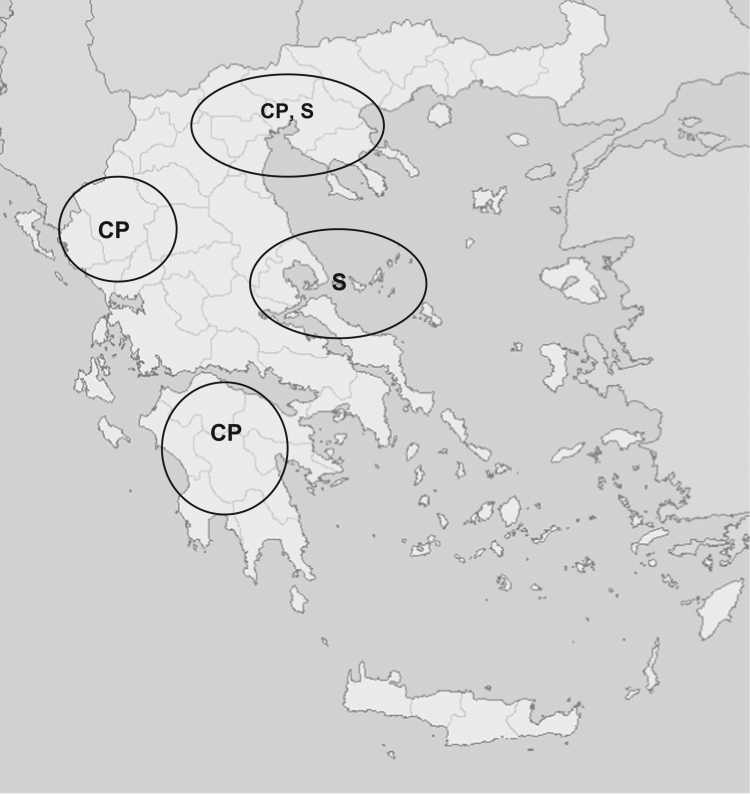
Geographical distribution of Greek goat flocks used for milk sample collection and proteomic analysis. The two breeds of indigenous Greek goats (CP: *Capra prisca*, S: Skopelos) included in the study can be seen scattered throughout Greece.

**Fig. 2:**
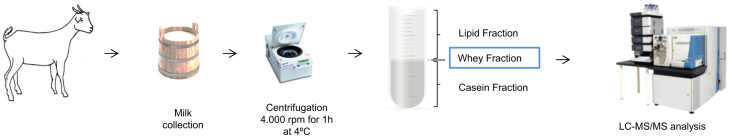
Flowchart of the strategy followed for analysis of milk. Following sample preparation the milk whey proteome was analyzed by nanoLC-MS/MS.

In order to obtain the most representative dataset as well as to eliminate any regional effect on the milk of two pure Greek goat breeds (*Capra prisca* and Skopelos), animals from flocks across Greece were analyzed. The geographical distribution of animal flocks used for milk sample collection is shown in Fig. 1. The flowchart of the strategy followed including the end-process for protein identification approaches used is schematically shown in Fig. 2. A total of 822 proteins were identified in the analyzed goat milk samples (Table 1). In Table 1 identified proteins are shown by their accession number and their description according to Uniprot database.

## 2 Experimental design, materials and methods

### 2.1 Animals and sample collection

Approximately 20 mL of milk collected from the two indigenous goat breed (*Capra prisca* and Skopelos), aged 3 to 5 years and 49 to 63 kg in weight. Immediately after collection, milk samples were centrifuged at 4000×*g* at 4 °C for 15 min, and the resulting fat layer was removed. The skim milk was transferred to sterile 1.5 mL microcentrifuge tubes and frozen at −20 °C until further analysis. All animal procedures regarding animal care and use were approved by the Ethical Committee of the Faculty of Veterinary Medicine, School of Health Sciences, University of Thessaly (Karditsa, Greece).

### 2.2 Sample preparation

Following complete thawing in room temperature, samples were centrifuged at 4000×*g* for 1 h at 4 °C, for final fragmentation in three layers (lipid-, whey-, casein-layer). The whey fraction was extracted and the protein content was determined the Bradford assay [2]. Whey fractions from all milk samples were routinely analyzed by nanoHPLC-MS/MS.

### 2.3 Peptide generation and 1-D nanoLC-MS/MS analysis

Protein extraction and peptide generation, was done as described by our group elsewhere, with few modifications [3]. In brief, whey fractions at a concentration of 200 ng were treated with 7 M urea buffer and 80 mM triethyl ammonium bicarbonate (TEAB) under mild sonication in a water-bath for 30 min. Reduction and alkylation steps of proteins were carried out using dithiothreitol and iodoacetamide solutions, at concentrations of 10 mM and 55 mM, respectively. The final step of processing included tryptic digestion of extracted proteins for peptide generation. Trypsin (Roche Diagnostics, Basel, Swiss) at a final concentration of 500 ng/μl, was applied to all samples in a humidified atmosphere, and samples were left to digest overnight.

### 2.4 LC-MS/MS analysis

Digested samples were analyzed using a LTQ Orbitrap Elite coupled to a Dionex 3000 nanoHPLC system (Thermo Scientific, Rockford, IL, USA). LC separation of peptides took place on two Thermo Scientific columns (PepMap® RSLC, C18, 100 Å, 3 μm-bead-packed 15 cm column and 2 μm-bead-packed 50 cm column) at a flow rate of 300 nL/min. The mobile phases A and B were 0.1% formic acid in water and 99% ACN in water, respectively. The gradient elution profile was as follows: 2.0% B (98.0% A) for 10 min, 2.0–35.0% B (98.0–65.0% A) for 325 min, 80.0% B (20.0% A) for 10 min, 2.0% B (98.0% A) for 10 min. Data were collected in the data-dependent MS/MS mode using a standard top-20 method. Full-scan data were acquired at a resolving power of 60,000 with a maximum integration time of 250 ms. Scan range was fixed at 250 to 1250 m/z and peptide fragmentation was performed in a higher-energy collision dissociation (HCD) mode with a normalized collision energy of 36%. MS/MS spectra were acquired with 15,000 resolving power and a maximum integration time of 120 ms. Measurements were performed using *m*/*z* 445.120025 as lock mass. Dynamic exclusion settings were set to repeat count 1, repeat duration 30 s, exclusion duration 120 s, and exclusion mass width 0.6 *m*/z (low) and 1.6 *m*/*z* (high).

The *.raw data files were analyzed using the Proteome Discoverer software (Thermo Scientific), using the Sequest search engine applying the *Ruminantae* for milk of goat *.fasta databases. MS/MS searches were performed using a 10 ppm parent ion mass tolerance and a 0.05 fragment mass tolerance. Trypsin was selected as the cleavage enzyme with up to 2 missed cleavage points. Cysteine methylthio modification was selected as a fixed modification and oxidation of methionine were selected as a variable. Peptide identifications were considered valid at 1% False Discovery Rate (*q*-value<0.01) (percolator maximum Delta Cn was 0.05). The minimum length of acceptable identified peptides was set as 6 amino acids.

## Footnotes

Appendix A Supplementary data associated with this article can be found in the online version at doi:doi:10.1016/j.dib.2016.06.038.
