# Supplementary material for: Dataset of milk whey proteins of two indigenous greek goat breeds
Source: Data Brief. 2016 Jun 28;8:692–6. doi: 10.1016/j.dib.2016.06.038 (PMC4949810; doi:10.1016/j.dib.2016.06.038)
Supplement: Supplementary file 1 — Transparency document [file mmc1.docx]

Conflicts of Interest

The authors declare that they have no Conflicts of Interest with regard to this Data in Brief article.

On behalf of the authors


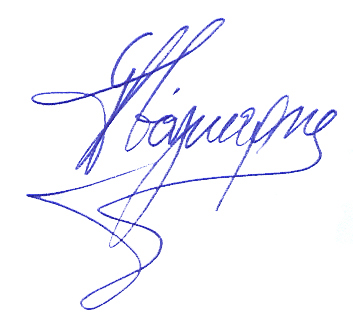


George Th. Tsangaris
