# Supplementary material for: Dataset of milk whey proteins of two indigenous greek goat breeds
Source: Data Brief. 2016 Jun 28;8:692–6. doi: 10.1016/j.dib.2016.06.038 (PMC4949810; doi:10.1016/j.dib.2016.06.038)
Supplement: Supplementary file 2 — Supplementary material [file mmc2.pdf]

Table 1. Proteins identified in Capra *prisca* (CP) and Skopelos (S) goat breeds

Accession	Description	Breed	
A0A068J685	NADH-ubiquinone oxidoreductase chain 5 OS=Bos mutus grunniens GN=ND5 PE=3 SV=1 - [A0A068J685_BOSMU]	CP	
A0A075T9L7	Dipeptidylpeptidase 4 OS=Ovis aries GN=DPP4 PE=2 SV=1 - [A0A075T9L7_SHEEP]	CP	
A0A088CHX5	ATP synthase subunit a OS=Capreolus capreolus GN=ATP6 PE=4 SV=1 - [A0A088CHX5_CAPCA]	CP	
A0A088QFM6	Heat shock protein 90kDa alpha OS=Bos mutus grunniens PE=2 SV=1 - [A0A088QFM6_BOSMU]	CP	
A0A0A7MB97	Nuclear factor erythroid 2-related factor 2 OS=Capra hircus GN=NFE2L2 PE=2 SV=1 - [A0A0A7MB97_CAPHI]	CP	
A0A0A7UX81	Cathelicidin 1 (Fragment) OS=Bubalus bubalis GN=CATHL1 PE=4 SV=1 - [A0A0A7UX81_BUBBU]	CP	
A0A0D3RIW8	Beta-casein (Fragment) OS=Bubalus bubalis GN=CSN2 PE=4 SV=1 - [A0A0D3RIW8_BUBBU]	CP	S
A0A0D5CBR5	Complement component 3 OS=Bubalus bubalis GN=C3 PE=2 SV=1 - [A0A0D5CBR5_BUBBU]	CP	S
A0A0D5MDG4	Kappa casein (Fragment) OS=Bubalus bubalis PE=4 SV=1 - [A0A0D5MDG4_BUBBU]		S
A0A0F6T485	Complement component 5 OS=Bubalus bubalis GN=C5 PE=2 SV=1 - [A0A0F6T485_BUBBU]		S
A0FEQ6	Myostatin (Fragment) OS=Ovis aries PE=3 SV=1 - [A0FEQ6_SHEEP]	CP	S
A0FH35	L-lactate dehydrogenase OS=Bos mutus grunniens PE=2 SV=1 - [A0FH35_BOSMU]	CP	S
A0FI82	Lipoprotein lipase OS=Capra hircus GN=LPL PE=2 SV=1 - [A0FI82_CAPHI]	CP	S
A0SWQ7	Kappa casein (Fragment) OS=Capra hircus GN=CSN3 PE=4 SV=1 - [A0SWQ7_CAPHI]	CP	
A1A4M4	Putative deoxyribonuclease TATDN3 OS=Bos taurus GN=TATDN3 PE=2 SV=1 - [TATD3_BOVIN]	CP	
A1A4M8	Torsin family 3, member A OS=Bos taurus GN=TOR3A PE=2 SV=1 - [A1A4M8_BOVIN]	CP	
A1L4Z4	RAD54-like protein OS=Bos taurus GN=RAD54L PE=2 SV=1 - [A1L4Z4_BOVIN]	CP	
A1XE97	Alpha 1 actinin (Fragment) OS=Bos taurus GN=ACTN1 PE=2 SV=1 - [A1XE97_BOVIN]	CP	
A1XEA7	Smooth muscle and non-muscle myosin alkali light chain peptide 6 (Fragment) OS=Bos taurus GN=MYL6 PE=2 SV=1 - [A1XEA7_BOVIN]	CP	S
A1YZ34	Xanthine oxidoreductase OS=Capra hircus PE=2 SV=1 - [A1YZ34_CAPHI]	CP	S
A2I7N2	Serpin A3-6 OS=Bos taurus GN=SERPINA3-6 PE=3 SV=1 - [SPA36_BOVIN]	CP	S
A2P2H1	VH region (Fragment) OS=Ovis aries GN=VH PE=2 SV=1 - [A2P2H1_SHEEP]		S
A2VE01	Polyribonucleotide 5'-hydroxyl-kinase Clp1 OS=Bos taurus GN=CLP1 PE=2 SV=1 - [CLP1_BOVIN]		S
A2VE03	ITCH protein OS=Bos taurus GN=ITCH PE=2 SV=1 - [A2VE03_BOVIN]	CP	
A3EY52	Butyrophilin subfamily 1 member A1 OS=Capra hircus GN=BTN1A1 PE=2 SV=1 - [A3EY52_CAPHI]	CP	S
A3F9D6	Lactoperoxidase OS=Capra hircus GN=LPO PE=1 SV=1 - [A3F9D6_CAPHI]	CP	S
A3QPC1	Lactoferrin OS=Capra hircus PE=2 SV=1 - [A3QPC1_CAPHI]	CP	S
A4FUA8	F-actin-capping protein subunit alpha-1 OS=Bos taurus GN=CAPZA1 PE=2 SV=1 - [CAZA1_BOVIN]	CP	
A4FUG2	LASS1 protein OS=Bos taurus GN=LASS1 PE=2 SV=1 - [A4FUG2_BOVIN]		S
A4IFJ3	MGC151592 protein OS=Bos taurus GN=MGC151592 PE=2 SV=1 - [A4IFJ3_BOVIN]	CP	S
A4ZVY6	Beta-2-microglobulin OS=Ovis aries PE=2 SV=1 - [A4ZVY6_SHEEP]	CP	
A5D785	Exportin-2 OS=Bos taurus GN=CSE1L PE=2 SV=1 - [XPO2_BOVIN]	CP	
A5D7J6	CALR protein OS=Bos taurus GN=CALR PE=1 SV=1 - [A5D7J6_BOVIN]	CP	
A5D7L9	NRAP protein OS=Bos taurus GN=NRAP PE=2 SV=1 - [A5D7L9_BOVIN]	CP	
A5D7Q9	OLFM3 protein OS=Bos taurus GN=OLFM3 PE=2 SV=1 - [A5D7Q9_BOVIN]	CP	S
A5D7S0	Alpha-soluble NSF attachment protein OS=Bos taurus GN=NAPA PE=2 SV=1 - [A5D7S0_BOVIN]	CP	S
A5D7U0	Cytoskeleton-associated protein 2 OS=Bos taurus GN=CKAP2 PE=2 SV=1 - [CKAP2_BOVIN]		S
A5D977	RALB protein OS=Bos taurus GN=RALB PE=2 SV=1 - [A5D977_BOVIN]		S
A5D984	Pyruvate kinase OS=Bos taurus GN=PKM2 PE=1 SV=1 - [A5D984_BOVIN]	CP	S
A5D9B6	Syntenin OS=Bos taurus GN=SDCBP PE=2 SV=1 - [A5D9B6_BOVIN]		S
A5D9D7	Procollagen-lysine, 2-oxoglutarate 5-dioxygenase 3 (Fragment) OS=Bos taurus GN=PLOD3 PE=2 SV=1 - [A5D9D7_BOVIN]	CP	
A5D9E1	Tumor protein D52 OS=Bos taurus GN=TPD52 PE=2 SV=1 - [A5D9E1_BOVIN]		S
A5JSR7	Beta-lactoglobulin (Fragment) OS=Capra hircus PE=2 SV=1 - [A5JSR7_CAPHI]	CP	S
A5JSS6	Procollagen-lysine 2-oxoglutarate 5-dioxygenase 3 (Fragment) OS=Capra hircus PE=2 SV=1 - [A5JSS6_CAPHI]		S
A5JSS7	Glycosylation-dependent cell adhesion molecule-1 OS=Capra hircus GN=GLYCAM1 PE=2 SV=1 - [A5JSS7_CAPHI]		S
A5JST2	Serum amyloid A protein OS=Capra hircus PE=2 SV=1 - [A5JST2_CAPHI]	CP	S
A5JV04	Stabilin-1 interacting chitinase-like protein (Fragment) OS=Ovis aries PE=2 SV=1 - [A5JV04_SHEEP]	CP	
A5PJA8	ER membrane protein complex subunit 7 OS=Bos taurus GN=EMC7 PE=2 SV=1 - [EMC7_BOVIN]		S
A5PK75	TOR1A protein OS=Bos taurus GN=TOR1A PE=2 SV=1 - [A5PK75_BOVIN]	CP	
A5YBU9	Complement factor B OS=Ovis aries GN=CFB PE=3 SV=1 - [A5YBU9_SHEEP]	CP	S
A6H7C2	Peroxisomal biogenesis factor 3 OS=Bos taurus GN=PEX3 PE=2 SV=1 - [PEX3_BOVIN]		S
A6H7J6	Protein disulfide-isomerase OS=Bos taurus GN=P4HB PE=1 SV=1 - [A6H7J6_BOVIN]		S
A6QL77	P4HA1 protein OS=Bos taurus GN=P4HA1 PE=2 SV=1 - [A6QL77_BOVIN]	CP	
A6QL80	PRMT3 protein OS=Bos taurus GN=PRMT3 PE=2 SV=1 - [A6QL80_BOVIN]	CP	
A6QLJ9	C22H3ORF18 protein OS=Bos taurus GN=C22H3ORF18 PE=2 SV=1 - [A6QLJ9_BOVIN]		S
A6QLL8	Fructose-bisphosphate aldolase OS=Bos taurus GN=ALDOA PE=1 SV=1 - [A6QLL8_BOVIN]	CP	S
A6QLS9	RAB10 protein OS=Bos taurus GN=RAB10 PE=1 SV=1 - [A6QLS9_BOVIN]	CP	
A6QM01	NAGLU protein OS=Bos taurus GN=NAGLU PE=2 SV=1 - [A6QM01_BOVIN]	CP	S
A6QM09	Putative uncharacterized protein OS=Bos taurus PE=2 SV=1 - [A6QM09_BOVIN]	CP	S
A6QNZ7	Keratin 10 (Epidermolytic hyperkeratosis; keratosis palmaris et plantaris) OS=Bos taurus GN=KRT10 PE=1 SV=1 - [A6QNZ7_BOVIN]	CP	S
A6QP36	LMAN2 protein OS=Bos taurus GN=LMAN2 PE=2 SV=1 - [A6QP36_BOVIN]	CP	S
A6QPD7	RNF121 protein OS=Bos taurus GN=RNF121 PE=2 SV=1 - [A6QPD7_BOVIN]	CP	
A6QPF2	FANCM protein OS=Bos taurus GN=FANCM PE=2 SV=1 - [A6QPF2_BOVIN]	CP	
A6QPM0	MGC148542 protein OS=Bos taurus GN=MGC148542 PE=2 SV=1 - [A6QPM0_BOVIN]	CP	
A6QPQ0	MORC4 protein OS=Bos taurus GN=MORC4 PE=2 SV=1 - [A6QPQ0_BOVIN]	CP	
A6QPR1	PCYOX1 protein OS=Bos taurus GN=PCYOX1 PE=2 SV=1 - [A6QPR1_BOVIN]	CP	S
A6QPY4	SGSH protein OS=Bos taurus GN=SGSH PE=2 SV=1 - [A6QPY4_BOVIN]	CP	
A6QQA8	Sulfhydryl oxidase OS=Bos taurus GN=QSOX1 PE=2 SV=1 - [A6QQA8_BOVIN]	CP	S
A6QQW5	C11H2ORF7 protein OS=Bos taurus GN=C11H2ORF7 PE=2 SV=1 - [A6QQW5_BOVIN]	CP	S
A6ZE97	Mannose-6-phosphate receptor binding protein 1 (Fragment) OS=Ovis aries GN=M6PRBP1 PE=2 SV=1 - [A6ZE97_SHEEP]		S
A6ZE99	Perilipin OS=Ovis aries GN=ADFP PE=2 SV=1 - [A6ZE99_SHEEP]	CP	S
A7MB21	FERMT2 protein OS=Bos taurus GN=FERMT2 PE=1 SV=1 - [A7MB21_BOVIN]		S
A7VJS3	Transient receptor potential cation channel, subfamily C, member 1, short isoform OS=Bos taurus GN=TRPC1 PE=2 SV=1 - [A7VJS3_BOVIN]		S
A7YW22	JAM3 protein OS=Bos taurus GN=JAM3 PE=2 SV=1 - [A7YW22_BOVIN]		S
A7YW91	PEX14 protein OS=Bos taurus GN=PEX14 PE=2 SV=1 - [A7YW91_BOVIN]		S
A8DBT6	Monocyte differentiation antigen CD14 OS=Bos taurus GN=CD14 PE=4 SV=1 - [A8DBT6_BOVIN]	CP	
A8E659	ANKH protein OS=Bos taurus GN=ANKH PE=2 SV=1 - [A8E659_BOVIN]		S
A9UIB1	PON1 OS=Bos taurus GN=PON1 PE=2 SV=1 - [A9UIB1_BOVIN]	CP	
A9YF36	Glyceraldehyde-3-phosphate dehydrogenase OS=Bos mutus grunniens GN=gapdh PE=2 SV=1 - [A9YF36_BOSMU]	CP	
A9YUB7	Osteopontin OS=Capra hircus GN=OPN PE=2 SV=1 - [A9YUB7_CAPHI]	CP	S
A9YY83	Beta casein (Fragment) OS=Bos indicus PE=4 SV=1 - [A9YY83_BOSIN]	CP	S
B0FZM7	Transaldolase 1 (Fragment) OS=Ovis aries PE=2 SV=1 - [B0FZM7_SHEEP]		S
B0JYQ0	ALB protein OS=Bos taurus GN=ALB PE=2 SV=1 - [B0JYQ0_BOVIN]	CP	
B0LRM8	Putative guanine nucleotide binding protein beta polypeptide 2-like 1 protein (Fragment) OS=Ovis aries PE=2 SV=1 - [B0LRM8_SHEEP]		S
B0LRN2	Serum amyloid A protein (Fragment) OS=Ovis aries PE=2 SV=1 - [B0LRN2_SHEEP]	CP	S

B1A4S9	Alpha-lactalbumin OS=Bos indicus PE=3 SV=1 - [B1A4S9_BOSIN]	CP	
B1A8Z2	Calcium and integrin-binding protein 1 OS=Ovis aries GN=CIB1 PE=2 SV=1 - [CIB1_SHEEP]	CP	S
B1PXC2	Cytochrome b (Fragment) OS=Odocoileus virginianus GN=cytb PE=4 SV=1 - [B1PXC2_ODOVR]	CP	
B2LSM5	Lipase OS=Ovis aries PE=2 SV=1 - [B2LSM5_SHEEP]	CP	
B2LSM6	Beta-hexosaminidase OS=Ovis aries PE=2 SV=1 - [B2LSM6_SHEEP]		S
B2LT73	Lactoferrin (Fragment) OS=Bubalus bubalis PE=4 SV=1 - [B2LT73_BUBBU]		S
B2LYK6	RAS oncogene family-like 7A OS=Ovis aries GN=RAB7A PE=2 SV=1 - [B2LYK6_SHEEP]		S
B2Z896	Kappa casein (Fragment) OS=Capra hircus PE=2 SV=1 - [B2Z896_CAPHI]	CP	S
B3VHM9	Albumin (Fragment) OS=Capra hircus PE=2 SV=1 - [B3VHM9_CAPHI]	CP	S
B3VSB9	ITM2B OS=Ovis aries GN=ITM2B PE=2 SV=1 - [B3VSB9_SHEEP]	CP	S
B5B0D4	Major allergen beta-lactoglobulin OS=Bos taurus PE=2 SV=1 - [B5B0D4_BOVIN]	CP	S
B5B3R8	Alpha S1 casein OS=Bos taurus GN=CSN1S1 PE=2 SV=1 - [B5B3R8_BOVIN]		S
B6D983	Alpha-1-acid glycoprotein OS=Capra ibex GN=AGP PE=2 SV=1 - [B6D983_CAPIB]	CP	
B6E141	Haptoglobin OS=Capra ibex GN=HP PE=2 SV=1 - [HPT_CAPIB]	CP	
B6UV62	SERPINF1 OS=Ovis aries GN=SERPINF1 PE=2 SV=1 - [B6UV62_SHEEP]	CP	S
B6ZBP2	Alpha S1 casein OS=Bubalus bubalis GN=CSN1S1 PE=2 SV=1 - [B6ZBP2_BUBBU]	CP	
B7TJ13	Phosphoglycerate kinase OS=Ovis aries GN=PGK1 PE=2 SV=1 - [B7TJ13_SHEEP]		S
B8QGZ0	Tumor necrosis factor receptor superfamily member 1B (Fragment) OS=Ovis aries GN=TNFRSF1B PE=2 SV=1 - [B8QGZ0_SHEEP]	CP	
B8R1K3	Transferrin OS=Bos mutus grunniens PE=2 SV=1 - [B8R1K3_BOSMU]	CP	S
B8Y9T0	Cumulus cell-specific fibronectin 1 transcript variant OS=Bos taurus GN=FN1 PE=1 SV=1 - [B8Y9T0_BOVIN]	CP	S
C4IXV8	Two pore channel 3 OS=Bos taurus GN=TPC3 PE=2 SV=1 - [C4IXV8_BOVIN]	CP	
C5IWU3	ADP-ribosylation factor 3 OS=Ovis aries GN=ARF3 PE=2 SV=1 - [C5IWU3_SHEEP]		S
C6KDV1	Toll-like receptor 7 (Fragment) OS=Ovis aries GN=TLR7 PE=4 SV=1 - [C6KDV1_SHEEP]		S
C6ZP47	I alpha globin OS=Ovis aries musimon GN=HBAI PE=3 SV=1 - [C6ZP47_OVIMU]	CP	S
C6ZP52	II alpha globin OS=Capra hircus GN=HBAII PE=3 SV=1 - [C6ZP52_CAPHI]	CP	
C7EDP2	Heat shock 70kDa protein 5 isoform 2 OS=Cervus elaphus GN=HSPA5 PE=2 SV=1 - [C7EDP2_CEREL]	CP	
C8BKC5	Peroxiredoxin 2 OS=Ovis aries GN=PRDX2 PE=2 SV=1 - [C8BKC5_SHEEP]	CP	
C8BKD1	Prothrombin OS=Ovis aries GN=F2 PE=2 SV=1 - [C8BKD1_SHEEP]	CP	
C8C9Q2	Keratin 5 (Fragment) OS=Ovis aries GN=KRT5 PE=2 SV=1 - [C8C9Q2_SHEEP]	CP	
D2DRB7	Alpha-s1-casein variant OS=Ovis aries GN=CSN1S1 PE=2 SV=1 - [D2DRB7_SHEEP]	CP	S
D2SZ86	K-casein (Fragment) OS=Ovis ammon collium GN=CSN3 PE=4 SV=1 - [D2SZ86_9CETA]		S
D2SZC8	K-casein (Fragment) OS=Ovis vignei arkal GN=CSN3 PE=4 SV=1 - [D2SZC8_OVIVI]	CP	S
D2U6V0	Alpha-1 acid glycoprotein OS=Bubalus bubalis GN=agp PE=2 SV=1 - [D2U6V0_BUBBU]		S
D6PVB9	Toll like receptor-2 (Fragment) OS=Capra hircus GN=TLR-2 PE=2 SV=1 - [D6PVB9_CAPHI]		S
D6PZY4	Factor H (Fragment) OS=Ovis aries GN=fH PE=2 SV=1 - [D6PZY4_SHEEP]		S
D7NJ85	Beta-casein OS=Bubalus bubalis GN=CSN2 PE=2 SV=2 - [D7NJ85_BUBBU]	CP	
D8X187	Serpin peptidase inhibitor clade B ovalbumin member 1 OS=Ovis aries GN=SERPINB1 PE=2 SV=1 - [D8X187_SHEEP]	CP	S
D9ZDE0	Chemokine (C-C motif) receptor 3 OS=Bos taurus PE=2 SV=1 - [D9ZDE0_BOVIN]	CP	
E1B748	Uncharacterized protein OS=Bos taurus GN=HYOU1 PE=1 SV=1 - [E1B748_BOVIN]	CP	
E1B7E3	Uncharacterized protein OS=Bos taurus GN=GOLGA4 PE=4 SV=2 - [E1B7E3_BOVIN]		S
E1B7Q4	Uncharacterized protein OS=Bos taurus GN=WDR19 PE=4 SV=2 - [E1B7Q4_BOVIN]		S
E1B7X1	Integrin-alpha FG-GAP repeat-containing protein 2 OS=Bos taurus GN=ITFG2 PE=4 SV=1 - [E1B7X1_BOVIN]	CP	
E1B8A0	Uncharacterized protein OS=Bos taurus GN=ACP5 PE=4 SV=1 - [E1B8A0_BOVIN]		S
E1B958	Uncharacterized protein OS=Bos taurus GN=ALMS1 PE=4 SV=2 - [E1B958_BOVIN]		S
E1B971	Uncharacterized protein OS=Bos taurus GN=RPTN PE=4 SV=2 - [E1B971_BOVIN]		S
E1B9H5	Uncharacterized protein OS=Bos taurus GN=TGFBR3 PE=4 SV=2 - [E1B9H5_BOVIN]	CP	S
E1BAZ1	Uncharacterized protein OS=Bos taurus GN=CLEC4A PE=4 SV=1 - [E1BAZ1_BOVIN]	CP	
E1BC41	Kinesin-like protein OS=Bos taurus GN=KIF15 PE=3 SV=2 - [E1BC41_BOVIN]		S
E1BC61	Uncharacterized protein OS=Bos taurus GN=PKDREJ PE=4 SV=2 - [E1BC61_BOVIN]	CP	
E1BC69	Uncharacterized protein OS=Bos taurus GN=HIPK3 PE=4 SV=2 - [E1BC69_BOVIN]		S
E1BC90	Uncharacterized protein OS=Bos taurus GN=CCNI2 PE=3 SV=1 - [E1BC90_BOVIN]		S
E1BD49	Uncharacterized protein (Fragment) OS=Bos taurus GN=FOXRED2 PE=4 SV=2 - [E1BD49_BOVIN]	CP	S
E1BDD3	Uncharacterized protein OS=Bos taurus GN=LOC618594 PE=4 SV=1 - [E1BDD3_BOVIN]	CP	
E1BF27	Uncharacterized protein OS=Bos taurus GN=SLC38A10 PE=1 SV=2 - [E1BF27_BOVIN]		S
E1BFS3	Uncharacterized protein OS=Bos taurus GN=SRRD PE=4 SV=2 - [E1BFS3_BOVIN]	CP	
E1BG25	Uncharacterized protein OS=Bos taurus GN=MFI2 PE=3 SV=1 - [E1BG25_BOVIN]	CP	S
E1BH06	Uncharacterized protein OS=Bos taurus GN=C4A PE=1 SV=2 - [E1BH06_BOVIN]	CP	S
E1BH45	Uncharacterized protein OS=Bos taurus GN=RB1CC1 PE=4 SV=1 - [E1BH45_BOVIN]	CP	
E1BHJ0	Profilin OS=Bos taurus PE=3 SV=1 - [E1BHJ0_BOVIN]	CP	S
E1BJB5	Uncharacterized protein OS=Bos taurus GN=NPHP4 PE=4 SV=2 - [E1BJB5_BOVIN]	CP	
E1BJN3	Amine oxidase OS=Bos taurus PE=3 SV=1 - [E1BJN3_BOVIN]		S
E1BJS1	Uncharacterized protein OS=Bos taurus GN=OGFOD3 PE=4 SV=1 - [E1BJS1_BOVIN]	CP	
E1BKW3	Uncharacterized protein OS=Bos taurus GN=MED23 PE=4 SV=2 - [E1BKW3_BOVIN]	CP	
E1BLR9	Uncharacterized protein OS=Bos taurus GN=CPD PE=4 SV=2 - [E1BLR9_BOVIN]	CP	
E1BM78	Uncharacterized protein OS=Bos taurus GN=FRMD3 PE=4 SV=1 - [E1BM78_BOVIN]	CP	
E1BM92	Uncharacterized protein OS=Bos taurus GN=PROM2 PE=4 SV=2 - [E1BM92_BOVIN]	CP	
E1BMG2	Uncharacterized protein OS=Bos taurus GN=DNAH5 PE=4 SV=2 - [E1BMG2_BOVIN]	CP	
E1BMJ0	Uncharacterized protein OS=Bos taurus GN=SERPING1 PE=3 SV=2 - [E1BMJ0_BOVIN]	CP	S
E1BND6	Uncharacterized protein (Fragment) OS=Bos taurus GN=LRBA PE=4 SV=2 - [E1BND6_BOVIN]		S
E1BNE2	Uncharacterized protein OS=Bos taurus GN=CAND2 PE=4 SV=2 - [E1BNE2_BOVIN]		S
E1BNI5	Uncharacterized protein OS=Bos taurus GN=SAMD15 PE=4 SV=1 - [E1BNI5_BOVIN]	CP	
E1BNW7	Uncharacterized protein OS=Bos taurus GN=ACOT12 PE=4 SV=2 - [E1BNW7_BOVIN]	CP	
E1BP81	Uncharacterized protein OS=Bos taurus GN=SEMA6A PE=4 SV=1 - [E1BP81_BOVIN]		S
E1BPD1	Olfactory receptor OS=Bos taurus GN=LOC532075 PE=3 SV=2 - [E1BPD1_BOVIN]	CP	
E1CEV8	Putative pheromone receptor sheepv1r1 (Fragment) OS=Ovis aries GN=sheepv1r1 PE=4 SV=1 - [E1CEV8_SHEEP]		S
E3VL24	Angiopoietin-like protein 4 (Fragment) OS=Capra hircus PE=2 SV=1 - [E3VL24_CAPHI]	CP	
E5EPD0	Glucose phosphate isomerase (Fragment) OS=Cervus elaphus hispanicus GN=GPI PE=2 SV=1 - [E5EPD0_CEREL]		S
E7BQS1	Alpha-S2-casein OS=Ovis aries GN=CSN1S2 PE=2 SV=1 - [E7BQS1_SHEEP]		S
E7BQS2	Alpha-s2-casein OS=Ovis aries GN=CSN1S2 PE=2 SV=1 - [E7BQS2_SHEEP]	CP	S
E7BQS5	Alpha-s2-casein OS=Ovis aries GN=CSN1S2 PE=2 SV=1 - [E7BQS5_SHEEP]	CP	
E7BSA0	TIMP metallopeptidase inhibitor (Fragment) OS=Tragelaphus eurycerus GN=TIMP3 PE=4 SV=1 - [E7BSA0_TRAEU]	CP	
E7BUN5	Leucyl/cystinyl aminopeptidase (Fragment) OS=Elaphurus davidianus GN=LNPEP PE=4 SV=1 - [E7BUN5_ELADA]		S
E7D7Z2	Osteopontin OS=Cervus nippon GN=OPN PE=2 SV=1 - [E7D7Z2_CERNI]		S
E7E1P5	Kappa casein (Fragment) OS=Bos indicus GN=CSN3 PE=4 SV=1 - [E7E1P5_BOSIN]		S
E9NRZ3	Beta-1,4-galactosyltransferase I OS=Capra hircus GN=B4GALT1 PE=2 SV=1 - [E9NRZ3_CAPHI]	CP	S
F1MB88	Uncharacterized protein (Fragment) OS=Bos taurus GN=ZBTB14 PE=4 SV=2 - [F1MB88_BOVIN]		S
F1MCP3	Uncharacterized protein OS=Bos taurus GN=BAZ2B PE=4 SV=2 - [F1MCP3_BOVIN]		S

F1MCR1	Uncharacterized protein OS=Bos taurus GN=LOC526041 PE=4 SV=2 - [F1MCR1_BOVIN]		S
F1MCX4	Tyrosine-protein kinase OS=Bos taurus GN=TYK2 PE=3 SV=2 - [F1MCX4_BOVIN]	CP	
F1MEN8	Protein disulfide-isomerase A4 OS=Bos taurus GN=PDIA4 PE=1 SV=1 - [F1MEN8_BOVIN]	CP	
F1MER7	Uncharacterized protein (Fragment) OS=Bos taurus GN=HSPG2 PE=4 SV=1 - [F1MER7_BOVIN]	CP	
F1MHA1	Uncharacterized protein (Fragment) OS=Bos taurus GN=KMT2A PE=4 SV=2 - [F1MHA1_BOVIN]	CP	
F1MJH1	Gelsolin OS=Bos taurus GN=GSN PE=1 SV=1 - [F1MJH1_BOVIN]		S
F1MJI0	Uncharacterized protein (Fragment) OS=Bos taurus GN=CCDC60 PE=4 SV=1 - [F1MJI0_BOVIN]	CP	
F1MJW4	Uncharacterized protein OS=Bos taurus GN=FAM20A PE=4 SV=2 - [F1MJW4_BOVIN]		S
F1MKF9	Uncharacterized protein OS=Bos taurus GN=LOC616308 PE=4 SV=2 - [F1MKF9_BOVIN]	CP	
F1MKS3	Uncharacterized protein (Fragment) OS=Bos taurus GN=TXNDC5 PE=1 SV=2 - [F1MKS3_BOVIN]	CP	S
F1ML32	Uncharacterized protein (Fragment) OS=Bos taurus GN=SCARF1 PE=4 SV=1 - [F1ML32_BOVIN]		S
F1MM31	Uncharacterized protein (Fragment) OS=Bos taurus GN=NCKAP5 PE=4 SV=2 - [F1MM31_BOVIN]	CP	
F1MM43	Uncharacterized protein OS=Bos taurus GN=RAD52 PE=4 SV=1 - [F1MM43_BOVIN]	CP	
F1MMP5	Inter-alpha-trypsin inhibitor heavy chain H1 OS=Bos taurus GN=ITIH1 PE=4 SV=1 - [F1MMP5_BOVIN]	CP	S
F1MPK6	Uncharacterized protein (Fragment) OS=Bos taurus GN=TNXB PE=4 SV=2 - [F1MPK6_BOVIN]	CP	
F1MQI1	Uncharacterized protein OS=Bos taurus GN=TLN2 PE=4 SV=2 - [F1MQI1_BOVIN]	CP	S
F1MS05	Aconitate hydratase OS=Bos taurus GN=ACO1 PE=1 SV=2 - [F1MS05_BOVIN]	CP	
F1MTP5	WD repeat-containing protein 1 OS=Bos taurus GN=WDR1 PE=4 SV=2 - [F1MTP5_BOVIN]		S
F1MTZ1	Protein NDRG2 OS=Bos taurus GN=NDRG2 PE=1 SV=2 - [F1MTZ1_BOVIN]	CP	
F1MU79	Peptidyl-prolyl cis-trans isomerase FKBP4 OS=Bos taurus GN=FKBP4 PE=1 SV=2 - [F1MU79_BOVIN]	CP	
F1MUX6	Uncharacterized protein (Fragment) OS=Bos taurus GN=GSTM3 PE=3 SV=2 - [F1MUX6_BOVIN]	CP	
F1MWB8	Uncharacterized protein (Fragment) OS=Bos taurus GN=DMXL2 PE=4 SV=2 - [F1MWB8_BOVIN]		S
F1MX92	Uncharacterized protein OS=Bos taurus GN=OR10K2 PE=4 SV=2 - [F1MX92_BOVIN]	CP	
F1MXJ5	IST1 homolog OS=Bos taurus GN=IST1 PE=4 SV=2 - [F1MXJ5_BOVIN]	CP	S
F1MXN4	Uncharacterized protein (Fragment) OS=Bos taurus GN=LRP11 PE=4 SV=2 - [F1MXN4_BOVIN]	CP	S
F1MYW3	Uncharacterized protein (Fragment) OS=Bos taurus GN=SEMA3E PE=4 SV=1 - [F1MYW3_BOVIN]	CP	
F1MZ26	Uncharacterized protein (Fragment) OS=Bos taurus PE=4 SV=2 - [F1MZ26_BOVIN]	CP	
F1MZJ5	Uncharacterized protein OS=Bos taurus GN=ADAM9 PE=4 SV=2 - [F1MZJ5_BOVIN]		S
F1MZU6	Collagen alpha-3(IV) chain OS=Bos taurus GN=COL4A3 PE=4 SV=2 - [F1MZU6_BOVIN]	CP	
F1MZV2	Uncharacterized protein OS=Bos taurus GN=CHMP5 PE=4 SV=1 - [F1MZV2_BOVIN]	CP	
F1N0A6	Uncharacterized protein OS=Bos taurus GN=ADGRV1 PE=4 SV=2 - [F1N0A6_BOVIN]	CP	
F1N1B8	Uncharacterized protein (Fragment) OS=Bos taurus GN=KANK1 PE=4 SV=1 - [F1N1B8_BOVIN]	CP	
F1N1W1	Oxygen-regulated protein 1 OS=Bos taurus GN=RP1 PE=4 SV=2 - [F1N1W1_BOVIN]	CP	
F1N272	Uncharacterized protein (Fragment) OS=Bos taurus GN=ATP13A4 PE=3 SV=1 - [F1N272_BOVIN]	CP	
F1N2I8	Uncharacterized protein OS=Bos taurus GN=RINL PE=4 SV=2 - [F1N2I8_BOVIN]		S
F1N334	Short-chain dehydrogenase/reductase 3 OS=Bos taurus GN=DHRS3 PE=3 SV=2 - [F1N334_BOVIN]	CP	
F1N3H1	Calumenin OS=Bos taurus GN=CALU PE=1 SV=1 - [F1N3H1_BOVIN]	CP	
F1N3J3	Uncharacterized protein OS=Bos taurus GN=AGR2 PE=1 SV=2 - [F1N3J3_BOVIN]	CP	S
F1N4Q7	Uncharacterized protein (Fragment) OS=Bos taurus GN=ARNTL2 PE=4 SV=2 - [F1N4Q7_BOVIN]		S
F1N4W9	Sodium channel protein (Fragment) OS=Bos taurus GN=SCN7A PE=3 SV=2 - [F1N4W9_BOVIN]	CP	
F1N554	Polypeptide N-acetylgalactosaminyltransferase (Fragment) OS=Bos taurus GN=GALNT10 PE=3 SV=2 - [F1N554_BOVIN]	CP	
F1N614	Uncharacterized protein OS=Bos taurus PE=3 SV=2 - [F1N614_BOVIN]		S
F1N650	Annexin OS=Bos taurus GN=ANXA1 PE=1 SV=1 - [F1N650_BOVIN]	CP	S
F1N6H1	Uncharacterized protein (Fragment) OS=Bos taurus GN=LRP2 PE=4 SV=2 - [F1N6H1_BOVIN]		S
F1N724	Uncharacterized protein OS=Bos taurus GN=DNAH11 PE=4 SV=2 - [F1N724_BOVIN]	CP	
F2Y4C9	BCL2-associated athanogene 3 (Fragment) OS=Elaphurus davidianus GN=BAG3 PE=4 SV=1 - [F2Y4C9_ELADA]		S
F2Y8N3	Storkhead box 2 (Fragment) OS=Elaphurus davidianus GN=STOX2 PE=4 SV=1 - [F2Y8N3_ELADA]		S
F2YQ13	Gelsolin isoform b OS=Ovis aries GN=GSN PE=2 SV=1 - [F2YQ13_SHEEP]	CP	
F4YD01	Aldehyde reductase (Fragment) OS=Bubalus bubalis GN=AKR1A1 PE=2 SV=1 - [F4YD01_BUBBU]	CP	
F4YD05	Calmodulin 2 (Fragment) OS=Bubalus bubalis GN=CALM2 PE=2 SV=1 - [F4YD05_BUBBU]		S
F4YD09	Superoxide dismutase [Cu-Zn] (Fragment) OS=Bubalus bubalis PE=2 SV=1 - [F4YD09_BUBBU]	CP	S
F6Q8Z3	Uncharacterized protein OS=Bos taurus GN=C11orf80 PE=4 SV=1 - [F6Q8Z3_BOVIN]	CP	
F6R6Q1	Uncharacterized protein OS=Bos taurus GN=FLAD1 PE=4 SV=1 - [F6R6Q1_BOVIN]		S
F6RM11	Uncharacterized protein OS=Bos taurus GN=NPTN PE=4 SV=1 - [F6RM11_BOVIN]		S
F8RKS1	1-acyl-sn-glycerol-3-phosphate acyltransferase OS=Capra hircus GN=LPAAT PE=2 SV=1 - [F8RKS1_CAPHI]	CP	S
F8U3U7	CD36 OS=Capra hircus GN=CD36 PE=2 SV=1 - [F8U3U7_CAPHI]	CP	S
G1DFV0	Oxysterol-binding protein OS=Capra hircus GN=OSBPL1A PE=2 SV=1 - [G1DFV0_CAPHI]		S
G3EHG5	Perilipin OS=Capra hircus PE=2 SV=1 - [G3EHG5_CAPHI]	CP	
G3MX97	Uncharacterized protein (Fragment) OS=Bos taurus GN=LOC101905441 PE=4 SV=1 - [G3MX97_BOVIN]		S
G3MXC8	Actin-related protein 2/3 complex subunit 5 OS=Bos taurus GN=ARPC5 PE=1 SV=1 - [G3MXC8_BOVIN]		S
G3MXM6	Uncharacterized protein OS=Bos taurus GN=AEBP1 PE=4 SV=1 - [G3MXM6_BOVIN]	CP	
G3MYH4	Tetraspanin (Fragment) OS=Bos taurus GN=CD81 PE=3 SV=1 - [G3MYH4_BOVIN]	CP	S
G3MYY9	Uncharacterized protein OS=Bos taurus GN=FAM83E PE=4 SV=1 - [G3MYY9_BOVIN]	CP	
G3MZ50	Uncharacterized protein (Fragment) OS=Bos taurus PE=4 SV=1 - [G3MZ50_BOVIN]	CP	
G3MZ88	Uncharacterized protein OS=Bos taurus GN=DNAJB9 PE=1 SV=1 - [G3MZ88_BOVIN]	CP	S
G3MZD6	Uncharacterized protein OS=Bos taurus GN=MAATS1 PE=4 SV=1 - [G3MZD6_BOVIN]	CP	
G3MZE2	Non-specific serine/threonine protein kinase OS=Bos taurus GN=PAK2 PE=1 SV=1 - [G3MZE2_BOVIN]	CP	
G3MZM2	Uncharacterized protein (Fragment) OS=Bos taurus PE=4 SV=1 - [G3MZM2_BOVIN]		S
G3MZT9	Uncharacterized protein (Fragment) OS=Bos taurus PE=3 SV=1 - [G3MZT9_BOVIN]		S
G3N0C7	Uncharacterized protein OS=Bos taurus PE=3 SV=1 - [G3N0C7_BOVIN]		S
G3N0I4	Cytosol aminopeptidase OS=Bos taurus GN=LAP3 PE=1 SV=1 - [G3N0I4_BOVIN]	CP	S
G3N0K1	Fermitin family homolog 3 OS=Bos taurus GN=FERMT3 PE=4 SV=1 - [G3N0K1_BOVIN]	CP	
G3N0X3	Uncharacterized protein OS=Bos taurus GN=IL17REL PE=4 SV=1 - [G3N0X3_BOVIN]		S
G3N139	Uncharacterized protein OS=Bos taurus GN=ZNF512B PE=4 SV=1 - [G3N139_BOVIN]	CP	
G3N152	Uncharacterized protein OS=Bos taurus GN=PCDHB8 PE=4 SV=1 - [G3N152_BOVIN]	CP	
G3N1L1	Uncharacterized protein OS=Bos taurus GN=LRRC10B PE=4 SV=1 - [G3N1L1_BOVIN]	CP	
G3N1Y5	Uncharacterized protein OS=Bos taurus GN=GPR85 PE=4 SV=1 - [G3N1Y5_BOVIN]		S
G3N2D7	Uncharacterized protein (Fragment) OS=Bos taurus GN=IGLL1 PE=4 SV=1 - [G3N2D7_BOVIN]	CP	S
G3N3N0	Uncharacterized protein (Fragment) OS=Bos taurus GN=ANK2 PE=4 SV=1 - [G3N3N0_BOVIN]		S
G3X742	Uncharacterized protein (Fragment) OS=Bos taurus GN=LOC530929 PE=4 SV=2 - [G3X742_BOVIN]	CP	
G3X7S2	Heat shock protein beta-1 OS=Bos taurus GN=HSPB1 PE=1 SV=1 - [G3X7S2_BOVIN]	CP	
G3X7S7	Tropomyosin alpha-1 chain (Fragment) OS=Bos taurus GN=TPM1 PE=1 SV=1 - [G3X7S7_BOVIN]	CP	
G3X8G9	Uncharacterized protein OS=Bos taurus PE=3 SV=1 - [G3X8G9_BOVIN]	CP	S
G5CC03	Milk fat globule EGF factor 8 protein (Fragment) OS=Capra hircus PE=2 SV=1 - [G5CC03_CAPHI]	CP	S
G5CWT9	Breast and ovarian cancer susceptibility 2 (Fragment) OS=Cervus nippon taiouanus GN=BRCA2 PE=4 SV=1 - [G5CWT9_CERNT]	CP	
G5E513	Uncharacterized protein (Fragment) OS=Bos taurus GN=IGHM PE=1 SV=1 - [G5E513_BOVIN]		S
G5E589	Proteasome subunit beta type OS=Bos taurus GN=PSMB1 PE=3 SV=1 - [G5E589_BOVIN]	CP	

G5E5D5	Uncharacterized protein OS=Bos taurus GN=LOC787476 PE=4 SV=1 - [G5E5D5_BOVIN]		S
G5E5T5	Uncharacterized protein (Fragment) OS=Bos taurus GN=IGHM PE=1 SV=1 - [G5E5T5_BOVIN]	CP	S
G5E6A5	Uncharacterized protein (Fragment) OS=Bos taurus PE=4 SV=1 - [G5E6A5_BOVIN]	CP	
G5E6K8	Uncharacterized protein (Fragment) OS=Bos taurus GN=LOC540057 PE=4 SV=1 - [G5E6K8_BOVIN]		S
G8FPL3	Kappa-casein (Fragment) OS=Nanger dama GN=KCAS PE=4 SV=1 - [G8FPL3_NANDA]	CP	S
G8IHT6	Alpha S1 casein (Fragment) OS=Bubalus bubalis GN=CSN1S1 PE=4 SV=1 - [G8IHT6_BUBBU]	CP	S
G9DAR3	Lactoferrin OS=Bubalus bubalis PE=2 SV=1 - [G9DAR3_BUBBU]	CP	S
H6UCL0	NADH-ubiquinone oxidoreductase chain 5 OS=Ourebia ourebi GN=ND5 PE=3 SV=1 - [H6UCL0_OUROU]		S
H9CH53	Beta-lactoglobulin (Fragment) OS=Bubalus bubalis GN=LGB PE=4 SV=1 - [H9CH53_BUBBU]	CP	S
I1WXR3	Alpha-1-antitrypsin transcript variant 1 OS=Ovis aries GN=SERPINA1 PE=2 SV=1 - [I1WXR3_SHEEP]	CP	S
I3Y1C6	Glutathione peroxidase (Fragment) OS=Bubalus bubalis PE=2 SV=1 - [I3Y1C6_BUBBU]		S
I6W7A2	Beta-actin OS=Capra hircus GN=ACTB PE=2 SV=1 - [I6W7A2_CAPHI]	CP	S
I6WY32	Alpha s1 casein OS=Capra hircus GN=CSN1S1 PE=2 SV=1 - [I6WY32_CAPHI]	CP	S
I6ZQY0	Heat shock cognate 71 kDa protein OS=Capra hircus GN=HSPA8 PE=2 SV=1 - [I6ZQY0_CAPHI]	CP	S
I7AL46	Guanine nucleotide binding protein beta polypeptide 2 (Fragment) OS=Ovis aries GN=GNB2 PE=2 SV=1 - [I7AL46_SHEEP]	CP	S
J7F5B7	Alpha-mannosidase OS=Capra hircus GN=MAN2B1 PE=2 SV=1 - [J7F5B7_CAPHI]	CP	S
J7H212	Keratin-associated protein KAP24-1 OS=Ovis aries GN=KRTAP24-1 PE=4 SV=1 - [J7H212_SHEEP]	CP	
K0P7C2	K-casein (Fragment) OS=Capra hircus GN=CSN3 PE=4 SV=1 - [K0P7C2_CAPHI]	CP	S
K4P494	Cystatin C OS=Ovis aries GN=CST3 PE=2 SV=1 - [K4P494_SHEEP]	CP	S
K8FK38	Heat Shock Protein 70 (Fragment) OS=Bos taurus GN=HSP70 PE=3 SV=1 - [K8FK38_BOVIN]	CP	S
L0BB45	Kappa casein (Fragment) OS=Ovis aries PE=4 SV=1 - [L0BB45_SHEEP]	CP	
L0CSP6	Peroxiredoxin-5 OS=Ovis aries GN=PRDX5 PE=2 SV=1 - [L0CSP6_SHEEP]	CP	
L8HKR7	Ig gamma-3 chain C region (Fragment) OS=Bos mutus GN=M91_08147 PE=4 SV=1 - [L8HKR7_9CETA]	CP	S
L8HLI3	Butyrophilin subfamily 1 member A1 OS=Bos mutus GN=M91_00347 PE=4 SV=1 - [L8HLI3_9CETA]	CP	S
L8HLX1	M-phase inducer phosphatase 1 (Fragment) OS=Bos mutus GN=M91_13687 PE=4 SV=1 - [L8HLX1_9CETA]		S
L8HMG3	Protein FAM32A OS=Bos mutus GN=M91_19156 PE=4 SV=1 - [L8HMG3_9CETA]		S
L8HMY2	Uncharacterized protein OS=Bos mutus GN=M91_03071 PE=4 SV=1 - [L8HMY2_9CETA]		S
L8HN56	Histone-lysine N-methyltransferase SETD2 (Fragment) OS=Bos mutus GN=M91_13698 PE=4 SV=1 - [L8HN56_9CETA]	CP	S
L8HNS1	Ankyrin repeat and SAM domain-containing protein 4B (Fragment) OS=Bos mutus GN=M91_06297 PE=4 SV=1 - [L8HNS1_9CETA]	CP	
L8HP71	Complement factor D (Fragment) OS=Bos mutus GN=M91_15533 PE=3 SV=1 - [L8HP71_9CETA]	CP	
L8HPF5	Ig gamma-1 chain C region (Fragment) OS=Bos mutus GN=M91_07836 PE=4 SV=1 - [L8HPF5_9CETA]		S
L8HQV0	Dehydrogenase/reductase SDR family member 1 OS=Bos mutus GN=M91_13225 PE=4 SV=1 - [L8HQV0_9CETA]	CP	S
L8HR13	Uncharacterized protein (Fragment) OS=Bos mutus GN=M91_17977 PE=4 SV=1 - [L8HR13_9CETA]		S
L8HRM3	Synaptic vesicle membrane protein VAT-1-like protein (Fragment) OS=Bos mutus GN=M91_15222 PE=4 SV=1 - [L8HRM3_9CETA]	CP	S
L8HRU6	Uncharacterized protein OS=Bos mutus GN=M91_17234 PE=4 SV=1 - [L8HRU6_9CETA]	CP	
L8HRW7	Uncharacterized protein (Fragment) OS=Bos mutus GN=M91_08728 PE=4 SV=1 - [L8HRW7_9CETA]	CP	S
L8HSP8	Uncharacterized protein OS=Bos mutus GN=M91_16939 PE=4 SV=1 - [L8HSP8_9CETA]	CP	
L8HSX3	Lysine-specific demethylase 5B (Fragment) OS=Bos mutus GN=M91_13310 PE=4 SV=1 - [L8HSX3_9CETA]	CP	
L8HU32	Uncharacterized protein (Fragment) OS=Bos mutus GN=M91_13456 PE=4 SV=1 - [L8HU32_9CETA]	CP	S
L8HU67	Protein Hook-like protein 1 OS=Bos mutus GN=M91_07358 PE=4 SV=1 - [L8HU67_9CETA]		S
L8HUK2	Taste receptor type 2 OS=Bos mutus GN=M91_21171 PE=3 SV=1 - [L8HUK2_9CETA]	CP	
L8HUM0	RING finger protein 160 OS=Bos mutus GN=M91_08990 PE=4 SV=1 - [L8HUM0_9CETA]		S
L8HUN7	Rho guanine nucleotide exchange factor 26 (Fragment) OS=Bos mutus GN=M91_00483 PE=4 SV=1 - [L8HUN7_9CETA]	CP	
L8HVF6	Selenoprotein M OS=Bos mutus GN=M91_11954 PE=4 SV=1 - [L8HVF6_9CETA]	CP	S
L8HVI9	Uncharacterized protein (Fragment) OS=Bos mutus GN=M91_08198 PE=4 SV=1 - [L8HVI9_9CETA]		S
L8HVY9	Hemopexin OS=Bos mutus GN=M91_19706 PE=3 SV=1 - [L8HVY9_9CETA]		S
L8HW76	Keratin, type II cytoskeletal 6A OS=Bos mutus GN=M91_10641 PE=3 SV=1 - [L8HW76_9CETA]		S
L8HXD3	Protein CDV3-like protein (Fragment) OS=Bos mutus GN=M91_17868 PE=4 SV=1 - [L8HXD3_9CETA]	CP	
L8HYQ8	Protein disulfide-isomerase OS=Bos mutus GN=M91_17341 PE=3 SV=1 - [L8HYQ8_9CETA]	CP	
L8HZ11	Tigger transposable element-derived protein 1 OS=Bos mutus GN=M91_16513 PE=4 SV=1 - [L8HZ11_9CETA]		S
L8I060	Fox-1-like protein A (Fragment) OS=Bos mutus GN=M91_18061 PE=4 SV=1 - [L8I060_9CETA]	CP	
L8I0D9	Semaphorin-7A (Fragment) OS=Bos mutus GN=M91_08455 PE=4 SV=1 - [L8I0D9_9CETA]	CP	
L8I173	KTEL motif-containing protein 1 OS=Bos mutus GN=M91_01380 PE=4 SV=1 - [L8I173_9CETA]	CP	
L8I1X5	Ellis-van Creveld syndrome protein (Fragment) OS=Bos mutus GN=M91_07780 PE=4 SV=1 - [L8I1X5_9CETA]		S
L8I250	Protein TANC1 (Fragment) OS=Bos mutus GN=M91_10222 PE=4 SV=1 - [L8I250_9CETA]	CP	
L8I2E5	DNA-binding protein inhibitor ID-3 OS=Bos mutus GN=M91_02240 PE=4 SV=1 - [L8I2E5_9CETA]	CP	
L8I2G1	Cell division control protein 42-like protein (Fragment) OS=Bos mutus GN=M91_02258 PE=3 SV=1 - [L8I2G1_9CETA]	CP	S
L8I2K0	Protein FAM124A (Fragment) OS=Bos mutus GN=M91_02375 PE=4 SV=1 - [L8I2K0_9CETA]		S
L8I2P6	Keratin, type I cytoskeletal 13 (Fragment) OS=Bos mutus GN=M91_00639 PE=3 SV=1 - [L8I2P6_9CETA]		S
L8I378	DnaJ-like protein subfamily C member 3 (Fragment) OS=Bos mutus GN=M91_06952 PE=4 SV=1 - [L8I378_9CETA]		S
L8I3C0	Histone H4 (Fragment) OS=Bos mutus GN=M91_09398 PE=3 SV=1 - [L8I3C0_9CETA]	CP	
L8I3E1	Olfactory receptor (Fragment) OS=Bos mutus GN=M91_04270 PE=3 SV=1 - [L8I3E1_9CETA]	CP	
L8I494	Globoside alpha-1,3-N-acetylgalactosaminyltransferase 1 OS=Bos mutus GN=M91_19123 PE=4 SV=1 - [L8I494_9CETA]		S
L8I5P8	Complement component C8 beta chain OS=Bos mutus GN=M91_03918 PE=4 SV=1 - [L8I5P8_9CETA]	CP	S
L8I5R0	Ceruloplasmin (Fragment) OS=Bos mutus GN=M91_03217 PE=4 SV=1 - [L8I5R0_9CETA]		S
L8I6I8	Chondroitin sulfate synthase 3 (Fragment) OS=Bos mutus GN=M91_01098 PE=4 SV=1 - [L8I6I8_9CETA]	CP	S
L8I7H2	UTP--glucose-1-phosphate uridylyltransferase (Fragment) OS=Bos mutus GN=M91_21508 PE=4 SV=1 - [L8I7H2_9CETA]	CP	
L8I868	Secretoglobin family 2A member 2 (Fragment) OS=Bos mutus GN=M91_15491 PE=4 SV=1 - [L8I868_9CETA]	CP	
L8I886	Peptidyl-prolyl cis-trans isomerase OS=Bos mutus GN=M91_02505 PE=3 SV=1 - [L8I886_9CETA]	CP	
L8I8A8	Malate dehydrogenase (Fragment) OS=Bos mutus GN=M91_21510 PE=3 SV=1 - [L8I8A8_9CETA]	CP	S
L8I8E0	Nck-associated protein 1-like protein OS=Bos mutus GN=M91_06018 PE=4 SV=1 - [L8I8E0_9CETA]	CP	
L8I8T5	Uncharacterized protein OS=Bos mutus GN=M91_04646 PE=3 SV=1 - [L8I8T5_9CETA]	CP	
L8I9Z4	Adenosylhomocysteinase (Fragment) OS=Bos mutus GN=M91_20171 PE=3 SV=1 - [L8I9Z4_9CETA]	CP	
L8IA28	Dedicator of cytokinesis protein 8 (Fragment) OS=Bos mutus GN=M91_20991 PE=4 SV=1 - [L8IA28_9CETA]	CP	
L8IAV6	Kell blood group glycoprotein (Fragment) OS=Bos mutus GN=M91_00621 PE=4 SV=1 - [L8IAV6_9CETA]		S
L8ICQ2	5'-nucleotidase (Fragment) OS=Bos mutus GN=M91_20600 PE=3 SV=1 - [L8ICQ2_9CETA]	CP	
L8ICR7	Ephrin-A1 (Fragment) OS=Bos mutus GN=M91_20862 PE=3 SV=1 - [L8ICR7_9CETA]	CP	S
L8IDB8	Polyubiquitin-C (Fragment) OS=Bos mutus GN=M91_18159 PE=4 SV=1 - [L8IDB8_9CETA]	CP	
L8IDD8	Uncharacterized protein OS=Bos mutus GN=M91_13886 PE=4 SV=1 - [L8IDD8_9CETA]		S
L8IEY9	Carboxypeptidase (Fragment) OS=Bos mutus GN=M91_17913 PE=3 SV=1 - [L8IEY9_9CETA]	CP	S
L8IF57	Beta-actin-like protein 2 OS=Bos mutus GN=M91_11587 PE=3 SV=1 - [L8IF57_9CETA]	CP	
L8IFD1	Protein FAM20A (Fragment) OS=Bos mutus GN=M91_10351 PE=4 SV=1 - [L8IFD1_9CETA]	CP	
L8IFF4	Nik-related protein kinase (Fragment) OS=Bos mutus GN=M91_07043 PE=4 SV=1 - [L8IFF4_9CETA]	CP	
L8IG28	Myosin regulatory light chain 2, ventricular/cardiac muscle isoform (Fragment) OS=Bos mutus GN=M91_16441 PE=4 SV=1 - [L8IG28_9CETA]		S
L8IG43	Vasculin OS=Bos mutus GN=M91_11585 PE=4 SV=1 - [L8IG43_9CETA]	CP	
L8IG53	Uncharacterized protein (Fragment) OS=Bos mutus GN=M91_11183 PE=4 SV=1 - [L8IG53_9CETA]		S
L8IG65	Oxysterols receptor LXR-alpha OS=Bos mutus GN=M91_11662 PE=3 SV=1 - [L8IG65_9CETA]	CP	

L8IG93	Glutathione peroxidase (Fragment) OS=Bos mutus GN=M91_14332 PE=3 SV=1 - [L8IG93_9CETA]	CP	S
L8IGH1	Copper transport protein ATOX1 (Fragment) OS=Bos mutus GN=M91_20718 PE=4 SV=1 - [L8IGH1_9CETA]	CP	
L8IGI5	C-X-C chemokine receptor type 5 OS=Bos mutus GN=M91_09726 PE=3 SV=1 - [L8IGI5_9CETA]		S
L8IGJ2	Secretory phospholipase A2 receptor (Fragment) OS=Bos mutus GN=M91_05747 PE=4 SV=1 - [L8IGJ2_9CETA]	CP	
L8IHE3	Cytokine receptor-like factor 2 (Fragment) OS=Bos mutus GN=M91_13493 PE=4 SV=1 - [L8IHE3_9CETA]	CP	
L8IHY7	von Willebrand factor A domain-containing protein 3A (Fragment) OS=Bos mutus GN=M91_00493 PE=4 SV=1 - [L8IHY7_9CETA]	CP	
L8IHZ5	MAM domain-containing protein 2 (Fragment) OS=Bos mutus GN=M91_01886 PE=4 SV=1 - [L8IHZ5_9CETA]	CP	
L8II39	Elongation factor 2 (Fragment) OS=Bos mutus GN=M91_12003 PE=4 SV=1 - [L8II39_9CETA]	CP	S
L8II70	Mediator of RNA polymerase II transcription subunit 1 OS=Bos mutus GN=M91_05272 PE=4 SV=1 - [L8II70_9CETA]		S
L8IIA4	Disheveled-associated activator of morphogenesis 2 OS=Bos mutus GN=M91_02424 PE=4 SV=1 - [L8IIA4_9CETA]		S
L8IIP1	Putative ATP-dependent RNA helicase DHX35 (Fragment) OS=Bos mutus GN=M91_02789 PE=4 SV=1 - [L8IIP1_9CETA]	CP	
L8IJG9	Chromatin assembly factor 1 subunit A OS=Bos mutus GN=M91_12018 PE=4 SV=1 - [L8IJG9_9CETA]	CP	
L8IJH2	Leucine-rich alpha-2-glycoprotein OS=Bos mutus GN=M91_12023 PE=4 SV=1 - [L8IJH2_9CETA]	CP	S
L8IJU8	Nucleobindin-1 OS=Bos mutus GN=M91_16014 PE=4 SV=1 - [L8IJU8_9CETA]	CP	S
L8IK69	Histone H2A OS=Bos mutus GN=M91_14430 PE=3 SV=1 - [L8IK69_9CETA]	CP	
L8IKI3	Eukaryotic initiation factor 4A-I (Fragment) OS=Bos mutus GN=M91_20379 PE=3 SV=1 - [L8IKI3_9CETA]	CP	
L8IKN8	T-cell immunomodulatory protein (Fragment) OS=Bos mutus GN=M91_10061 PE=4 SV=1 - [L8IKN8_9CETA]		S
L8IKS7	Tetranectin OS=Bos mutus GN=M91_08233 PE=4 SV=1 - [L8IKS7_9CETA]	CP	S
L8IL03	MICAL-like protein 1 (Fragment) OS=Bos mutus GN=M91_14186 PE=4 SV=1 - [L8IL03_9CETA]	CP	
L8ILD0	Uroplakin-3a OS=Bos mutus GN=M91_12338 PE=4 SV=1 - [L8ILD0_9CETA]		S
L8ILP6	Plasma serine protease inhibitor OS=Bos mutus GN=M91_13739 PE=3 SV=1 - [L8ILP6_9CETA]	CP	
L8IM91	60S acidic ribosomal protein P2 (Fragment) OS=Bos mutus GN=M91_00327 PE=3 SV=1 - [L8IM91_9CETA]	CP	S
L8ING2	A-kinase anchor protein 2 (Fragment) OS=Bos mutus GN=M91_02685 PE=4 SV=1 - [L8ING2_9CETA]	CP	
L8INH5	Annexin (Fragment) OS=Bos mutus GN=M91_09602 PE=3 SV=1 - [L8INH5_9CETA]	CP	
L8IP25	Aldehyde oxidase OS=Bos mutus GN=M91_14969 PE=4 SV=1 - [L8IP25_9CETA]	CP	S
L8IPI0	GATS-like protein 2 (Fragment) OS=Bos mutus GN=M91_01082 PE=4 SV=1 - [L8IPI0_9CETA]	CP	
L8IQ45	Protein OSCP1 OS=Bos mutus GN=M91_09554 PE=4 SV=1 - [L8IQ45_9CETA]	CP	
L8IQ75	Hypoxia-inducible factor 3-alpha (Fragment) OS=Bos mutus GN=M91_08187 PE=4 SV=1 - [L8IQ75_9CETA]		S
L8IQE7	Laminin subunit gamma-1 (Fragment) OS=Bos mutus GN=M91_14901 PE=4 SV=1 - [L8IQE7_9CETA]	CP	S
L8IQM1	Protein CREG1 (Fragment) OS=Bos mutus GN=M91_19211 PE=4 SV=1 - [L8IQM1_9CETA]	CP	S
L8IQV4	Dystroglycan OS=Bos mutus GN=M91_12443 PE=4 SV=1 - [L8IQV4_9CETA]		S
L8IR22	Basigin OS=Bos mutus GN=M91_01904 PE=4 SV=1 - [L8IR22_9CETA]		S
L8IR26	Folate receptor alpha OS=Bos mutus GN=M91_03529 PE=4 SV=1 - [L8IR26_9CETA]		S
L8IRG0	HEAT repeat-containing protein 4 (Fragment) OS=Bos mutus GN=M91_00658 PE=4 SV=1 - [L8IRG0_9CETA]	CP	
L8ISN2	Ephrin type-A receptor 10 OS=Bos mutus GN=M91_09542 PE=4 SV=1 - [L8ISN2_9CETA]		S
L8ISN7	Axonemal dynein light intermediate polypeptide 1 (Fragment) OS=Bos mutus GN=M91_09547 PE=4 SV=1 - [L8ISN7_9CETA]		S
L8ISV3	Alpha-enolase OS=Bos mutus GN=M91_12821 PE=3 SV=1 - [L8ISV3_9CETA]	CP	S
L8ISW4	6-phosphogluconate dehydrogenase, decarboxylating (Fragment) OS=Bos mutus GN=M91_12838 PE=3 SV=1 - [L8ISW4_9CETA]	CP	S
L8ITE5	Uncharacterized protein (Fragment) OS=Bos mutus GN=M91_02140 PE=4 SV=1 - [L8ITE5_9CETA]		S
L8ITR4	Calsyntenin-1 (Fragment) OS=Bos mutus GN=M91_12831 PE=4 SV=1 - [L8ITR4_9CETA]		S
L8ITX2	Protein FAM84A OS=Bos mutus GN=M91_00605 PE=4 SV=1 - [L8ITX2_9CETA]	CP	
L8IU67	Sortilin (Fragment) OS=Bos mutus GN=M91_01627 PE=4 SV=1 - [L8IU67_9CETA]	CP	
L8IWB3	Erythrocyte band 7 integral membrane protein (Fragment) OS=Bos mutus GN=M91_04259 PE=4 SV=1 - [L8IWB3_9CETA]	CP	
L8IWX7	Glutamate [NMDA] receptor subunit epsilon-1 (Fragment) OS=Bos mutus GN=M91_04832 PE=4 SV=1 - [L8IWX7_9CETA]	CP	
L8IX03	Pituitary adenylate cyclase-activating polypeptide type I receptor OS=Bos mutus GN=M91_19648 PE=4 SV=1 - [L8IX03_9CETA]	CP	
L8IX69	Low-density lipoprotein receptor (Fragment) OS=Bos mutus GN=M91_04910 PE=4 SV=1 - [L8IX69_9CETA]	CP	
L8IY78	EF-hand domain-containing protein D2 (Fragment) OS=Bos mutus GN=M91_10961 PE=4 SV=1 - [L8IY78_9CETA]	CP	S
L8IYQ4	UDP-glucuronic acid decarboxylase 1 (Fragment) OS=Bos mutus GN=M91_19261 PE=4 SV=1 - [L8IYQ4_9CETA]	CP	S
L8IZ21	Pigment epithelium-derived factor OS=Bos mutus GN=M91_14691 PE=3 SV=1 - [L8IZ21_9CETA]	CP	S
L8IZU2	Uncharacterized protein (Fragment) OS=Bos mutus GN=M91_02858 PE=4 SV=1 - [L8IZU2_9CETA]	CP	
L8J0I1	Renin receptor (Fragment) OS=Bos mutus GN=M91_17207 PE=4 SV=1 - [L8J0I1_9CETA]	CP	S
L8J0J3	Armadillo repeat-containing protein 4 OS=Bos mutus GN=M91_00322 PE=4 SV=1 - [L8J0J3_9CETA]		S
L8J0J5	Uncharacterized protein OS=Bos mutus GN=M91_14931 PE=3 SV=1 - [L8J0J5_9CETA]	CP	
L8J0J6	Poly(A) RNA polymerase, mitochondrial (Fragment) OS=Bos mutus GN=M91_21095 PE=4 SV=1 - [L8J0J6_9CETA]	CP	
L8J0K4	Platelet endothelial cell adhesion molecule (Fragment) OS=Bos mutus GN=M91_08946 PE=4 SV=1 - [L8J0K4_9CETA]		S
L8J0N4	Pleckstrin-like protein domain-containing family J member 1 OS=Bos mutus GN=M91_18665 PE=4 SV=1 - [L8J0N4_9CETA]	CP	
L8J0R5	Relaxin-3 receptor 1 (Fragment) OS=Bos mutus GN=M91_05508 PE=4 SV=1 - [L8J0R5_9CETA]	CP	
L8J0V2	Proteasome subunit alpha type (Fragment) OS=Bos mutus GN=M91_07663 PE=3 SV=1 - [L8J0V2_9CETA]		S
L8J119	Thrombospondin-1 (Fragment) OS=Bos mutus GN=M91_12734 PE=4 SV=1 - [L8J119_9CETA]	CP	S
L8J2M8	14-3-3 protein epsilon (Fragment) OS=Bos mutus GN=M91_14679 PE=3 SV=1 - [L8J2M8_9CETA]	CP	S
L8J3F8	Integral membrane protein 2C (Fragment) OS=Bos mutus GN=M91_11017 PE=4 SV=1 - [L8J3F8_9CETA]		S
L8J3H9	14-3-3 protein zeta/delta (Fragment) OS=Bos mutus GN=M91_19634 PE=4 SV=1 - [L8J3H9_9CETA]	CP	
L8J3U6	JmjC domain-containing protein 8 (Fragment) OS=Bos mutus GN=M91_08791 PE=4 SV=1 - [L8J3U6_9CETA]	CP	
L8J3X7	Dynein heavy chain 7, axonemal (Fragment) OS=Bos mutus GN=M91_08599 PE=4 SV=1 - [L8J3X7_9CETA]		S
L8J6A1	Endothelial lipase OS=Bos mutus GN=M91_10240 PE=3 SV=1 - [L8J6A1_9CETA]		S
M1RB49	Alpha-lactalbumin (Fragment) OS=Bubalus bubalis GN=aLA PE=4 SV=1 - [M1RB49_BUBBU]		S
O46390	Biglycan OS=Ovis aries GN=BGN PE=2 SV=1 - [PGS1_SHEEP]	CP	S
O46544	Complement component C3 (Fragment) OS=Ovis aries GN=C3 PE=2 SV=1 - [O46544_SHEEP]	CP	S
O62826	Short transient receptor potential channel 2 homolog OS=Bos taurus GN=TRPC2 PE=2 SV=1 - [TRPC2_BOVIN]	CP	
O77777	Beta-lactoglobulin variant D (Fragment) OS=Bos taurus GN=LGB PE=4 SV=1 - [O77777_BOVIN]	CP	S
O97802	Latrophilin 2 splice variant baaae OS=Bos taurus PE=2 SV=1 - [O97802_BOVIN]	CP	
P00745	Vitamin K-dependent protein C (Fragment) OS=Bos taurus GN=PROC PE=1 SV=1 - [PROC_BOVIN]		S
P01131	Low-density lipoprotein receptor (Fragment) OS=Bos taurus GN=LDLR PE=2 SV=1 - [LDLR_BOVIN]		S
P02076	Hemoglobin subunit beta OS=Ovis aries musimon GN=HBB PE=1 SV=1 - [HBB_OVIMU]		S
P02077	Hemoglobin subunit beta-A OS=Capra hircus PE=1 SV=1 - [HBBA_CAPHI]	CP	
P04272	Annexin A2 OS=Bos taurus GN=ANXA2 PE=1 SV=2 - [ANXA2_BOVIN]		S
P08728	Keratin, type I cytoskeletal 19 OS=Bos taurus GN=KRT19 PE=2 SV=1 - [K1C19_BOVIN]	CP	S
P0DMA8	Apolipoprotein A-I OS=Pantholops hodgsonii GN=APOA1 PE=2 SV=1 - [APOA1_PANHO]		S
P11839	Beta-casein OS=Ovis aries GN=CSN2 PE=1 SV=3 - [CASB_SHEEP]	CP	
P12303	Transthyretin OS=Ovis aries GN=TTR PE=2 SV=1 - [TTHY_SHEEP]	CP	S
P15103	Glutamine synthetase OS=Bos taurus GN=GLUL PE=2 SV=4 - [GLNA_BOVIN]		S
P15497	Apolipoprotein A-I OS=Bos taurus GN=APOA1 PE=1 SV=3 - [APOA1_BOVIN]	CP	
P17248	Tryptophan--tRNA ligase, cytoplasmic OS=Bos taurus GN=WARS PE=1 SV=3 - [SYWC_BOVIN]		S
P17697	Clusterin OS=Bos taurus GN=CLU PE=1 SV=1 - [CLUS_BOVIN]	CP	
P18626	Alpha-S1-casein OS=Capra hircus GN=CSN1S1 PE=1 SV=2 - [CASA1_CAPHI]	CP	S
P29701	Alpha-2-HS-glycoprotein OS=Ovis aries GN=AHSG PE=1 SV=1 - [FETUA_SHEEP]	CP	S
P32262	Antithrombin-III OS=Ovis aries GN=SERPINC1 PE=2 SV=1 - [ANT3_SHEEP]	CP	S

P33048	Beta-casein OS=Capra hircus GN=CSN2 PE=2 SV=1 - [CASB_CAPHI]	CP	S
P33049	Alpha-S2-casein OS=Capra hircus GN=CSN1S2 PE=2 SV=1 - [CASA2_CAPHI]	CP	S
P34955	Alpha-1-antiproteinase OS=Bos taurus GN=SERPINA1 PE=1 SV=1 - [A1AT_BOVIN]	CP	
P37980	Inorganic pyrophosphatase OS=Bos taurus GN=PPA1 PE=1 SV=2 - [IPYR_BOVIN]	CP	
P42819	Serum amyloid A protein OS=Ovis aries GN=SAA1 PE=1 SV=1 - [SAA_SHEEP]	CP	
P42917	Inhibin beta B chain OS=Bos taurus GN=INHBB PE=3 SV=1 - [INHBB_BOVIN]		S
P48644	Retinal dehydrogenase 1 OS=Bos taurus GN=ALDH1A1 PE=2 SV=3 - [AL1A1_BOVIN]	CP	S
P50450	Thyroxine-binding globulin OS=Ovis aries GN=SERPINA7 PE=2 SV=1 - [THBG_SHEEP]	CP	
P61585	Transforming protein RhoA OS=Bos taurus GN=RHOA PE=1 SV=1 - [RHOA_BOVIN]		S
P67975	Beta-lactoglobulin OS=Ovis aries musimon GN=LGB PE=1 SV=1 - [LACB_OVIMU]	CP	S
P68215	Fibrinogen alpha chain (Fragment) OS=Capra hircus GN=FGA PE=1 SV=1 - [FIBA_CAPHI]	CP	S
P68251	14-3-3 protein beta/alpha (Fragments) OS=Ovis aries GN=YWHAB PE=1 SV=2 - [1433B_SHEEP]	CP	
P79362	Cathelicidin-2 OS=Ovis aries GN=CATHL2 PE=2 SV=1 - [CTHL2_SHEEP]	CP	S
P80311	Peptidyl-prolyl cis-trans isomerase B OS=Bos taurus GN=PPIB PE=1 SV=4 - [PPIB_BOVIN]	CP	
P81447	Glycosylation-dependent cell adhesion molecule 1 OS=Capra hircus GN=GLYCAM1 PE=1 SV=2 - [GLCM1_CAPHI]	CP	
Q06B57	Fatty acid synthase OS=Capra hircus GN=FASN PE=2 SV=2 - [Q06B57_CAPHI]	CP	
Q076H7	Fatty acid synthase OS=Capra hircus GN=FASN PE=2 SV=1 - [Q076H7_CAPHI]	CP	S
Q08DD6	Interferon regulatory factor 6 OS=Bos taurus GN=IRF6 PE=2 SV=1 - [IRF6_BOVIN]	CP	
Q08DP0	Phosphoglucomutase-1 OS=Bos taurus GN=PGM1 PE=2 SV=1 - [PGM1_BOVIN]	CP	
Q08E20	S-formylglutathione hydrolase OS=Bos taurus GN=ESD PE=2 SV=1 - [ESTD_BOVIN]	CP	S
Q0IIG8	Ras-related protein Rab-18 OS=Bos taurus GN=RAB18 PE=2 SV=1 - [RAB18_BOVIN]	CP	S
Q0P587	Glucose-6-phosphate 1-dehydrogenase (Fragment) OS=Bos taurus GN=H6PD PE=2 SV=1 - [Q0P587_BOVIN]		S
Q0P5I0	Menin OS=Bos taurus GN=MEN1 PE=2 SV=1 - [MEN1_BOVIN]		S
Q0PEU2	Keratin 14 (Fragment) OS=Bubalus bubalis PE=2 SV=1 - [Q0PEU2_BUBBU]	CP	
Q0PGG4	Actin, cytoplasmic 1 OS=Bos mutus grunniens GN=ACTB PE=2 SV=1 - [ACTB_BOSMU]		S
Q0V8B5	BCSC-1 isoform 1 (Fragment) OS=Bos taurus GN=LOH11CR2A PE=2 SV=1 - [Q0V8B5_BOVIN]	CP	
Q0V8Q0	Discoidin domain receptor family, member 1 (Fragment) OS=Bos taurus GN=DDR1 PE=2 SV=1 - [Q0V8Q0_BOVIN]	CP	S
Q148C9	Heme-binding protein 1 OS=Bos taurus GN=HEBP1 PE=2 SV=1 - [HEBP1_BOVIN]	CP	S
Q148J6	Actin-related protein 2/3 complex subunit 4 OS=Bos taurus GN=ARPC4 PE=1 SV=3 - [ARPC4_BOVIN]		S
Q17QC7	Poliovirus receptor-related 2 (Herpesvirus entry mediator B) OS=Bos taurus GN=PVRL2 PE=2 SV=1 - [Q17QC7_BOVIN]	CP	
Q17QW3	Retinol dehydrogenase 14 (All-trans/9-cis/11-cis) OS=Bos taurus GN=RDH14 PE=2 SV=1 - [Q17QW3_BOVIN]	CP	S
Q1JPH2	Ras related v-ral simian leukemia viral oncogene homolog A (Fragment) OS=Bos taurus GN=RALA PE=2 SV=1 - [Q1JPH2_BOVIN]	CP	S
Q1PBC9	Monocyte differentiation antigen CD14 OS=Capra hircus PE=2 SV=1 - [Q1PBC9_CAPHI]	CP	S
Q1PHW0	Phosphatidylethanolamine-binding protein OS=Cervus elaphus PE=2 SV=1 - [Q1PHW0_CEREL]	CP	
Q1RMJ6	Rho-related GTP-binding protein RhoC OS=Bos taurus GN=RHOC PE=2 SV=1 - [RHOC_BOVIN]		S
Q1RMN0	Aspartylglucosaminidase OS=Bos taurus GN=AGA PE=2 SV=1 - [Q1RMN0_BOVIN]	CP	
Q1RMP3	CutA divalent cation tolerance homolog (E. coli) OS=Bos taurus GN=CUTA PE=2 SV=1 - [Q1RMP3_BOVIN]	CP	S
Q1XE70	Toll-like receptor 2 (Fragment) OS=Ovis aries GN=tlr2 PE=2 SV=1 - [Q1XE70_SHEEP]	CP	
Q28024	Guanine nucleotide-binding protein G(I)/G(S)/G(O) subunit gamma-12 OS=Bos taurus GN=GNG12 PE=1 SV=2 - [GBG12_BOVIN]	CP	S
Q28078	Epithelial mucin (Fragment) OS=Bos taurus GN=MUC1 PE=4 SV=1 - [Q28078_BOVIN]		S
Q28174	P21 ras protein (Fragment) OS=Bos taurus GN=p21 ras PE=4 SV=1 - [Q28174_BOVIN]		S
Q28554	Glyceraldehyde-3-phosphate dehydrogenase (Fragment) OS=Ovis aries GN=GAPDH PE=2 SV=4 - [G3P_SHEEP]		S
Q29RY4	Zinc phosphodiesterase ELAC protein 1 OS=Bos taurus GN=ELAC1 PE=2 SV=1 - [RNZ1_BOVIN]		S
Q2HJD0	FAS-associated factor 2 OS=Bos taurus GN=FAF2 PE=2 SV=1 - [FAF2_BOVIN]		S
Q2HJH2	Ras-related protein Rab-1B OS=Bos taurus GN=RAB1B PE=2 SV=1 - [RAB1B_BOVIN]	CP	S
Q2KHZ3	Metal response element binding transcription factor 2 OS=Bos taurus GN=MTF2 PE=2 SV=1 - [Q2KHZ3_BOVIN]	CP	
Q2KJF1	Alpha-1B-glycoprotein OS=Bos taurus GN=A1BG PE=1 SV=1 - [A1BG_BOVIN]	CP	S
Q2PZL1	Prolactin (Fragment) OS=Bubalus bubalis PE=4 SV=1 - [Q2PZL1_BUBBU]	CP	
Q2T9S1	CCPG1 protein (Fragment) OS=Bos taurus GN=CCPG1 PE=2 SV=1 - [Q2T9S1_BOVIN]	CP	
Q2T9V9	PHD finger protein 10 OS=Bos taurus GN=PHF10 PE=2 SV=1 - [PHF10_BOVIN]		S
Q2TBX4	Heat shock 70 kDa protein 13 OS=Bos taurus GN=HSPA13 PE=2 SV=1 - [HSP13_BOVIN]	CP	
Q2TJL4	Butyrophilin (Fragment) OS=Bos indicus PE=4 SV=1 - [Q2TJL4_BOSIN]	CP	
Q307G8	Ras-related protein RAB11A (Fragment) OS=Ovis aries PE=2 SV=1 - [Q307G8_SHEEP]	CP	
Q307G9	Cysteine-rich angiogenic inducer 61 (Fragment) OS=Ovis aries PE=2 SV=1 - [Q307G9_SHEEP]		S
Q30B76	Niemann-Pick disease type C2 (Fragment) OS=Ovis aries PE=2 SV=1 - [Q30B76_SHEEP]	CP	S
Q32KL2	Proteasome subunit beta type-5 OS=Bos taurus GN=PSMB5 PE=1 SV=1 - [PSB5_BOVIN]	CP	
Q3LRQ1	Vitronectin (Fragment) OS=Capra hircus PE=2 SV=1 - [Q3LRQ1_CAPHI]	CP	
Q3MHM5	Tubulin beta-4B chain OS=Bos taurus GN=TUBB4B PE=2 SV=1 - [TBB4B_BOVIN]	CP	S
Q3MHZ3	ATP6AP1 protein (Fragment) OS=Bos taurus GN=ATP6AP1 PE=2 SV=1 - [Q3MHZ3_BOVIN]	CP	S
Q3SX06	Myocilin OS=Bos taurus GN=MYOC PE=2 SV=1 - [Q3SX06_BOVIN]	CP	
Q3SYR8	Immunoglobulin J chain OS=Bos taurus GN=IGJ PE=1 SV=1 - [Q3SYR8_BOVIN]	CP	S
Q3SZ45	Stromal cell-derived factor 2 OS=Bos taurus GN=SDF2 PE=2 SV=1 - [SDF2_BOVIN]	CP	S
Q3SZH7	Leukotriene A-4 hydrolase OS=Bos taurus GN=LTA4H PE=2 SV=3 - [LKHA4_BOVIN]	CP	S
Q3SZI4	14-3-3 protein theta OS=Bos taurus GN=YWHAQ PE=2 SV=1 - [1433T_BOVIN]	CP	
Q3T000	Synaptobrevin homolog YKT6 OS=Bos taurus GN=YKT6 PE=2 SV=1 - [YKT6_BOVIN]	CP	S
Q3T0D7	GTP-binding protein SAR1a OS=Bos taurus GN=SAR1A PE=2 SV=1 - [SAR1A_BOVIN]		S
Q3T0E5	Adipocyte plasma membrane-associated protein OS=Bos taurus GN=APMAP PE=2 SV=1 - [APMAP_BOVIN]		S
Q3T0R2	NDUFS4 protein (Fragment) OS=Bos taurus GN=NDUFS4 PE=2 SV=1 - [Q3T0R2_BOVIN]	CP	
Q3T2K8	Caspase-15 (Fragment) OS=Ovis aries PE=2 SV=1 - [Q3T2K8_SHEEP]		S
Q3ZBA6	DnaJ homolog subfamily B member 11 OS=Bos taurus GN=DNAJB11 PE=2 SV=1 - [DJB11_BOVIN]	CP	S
Q3ZBV8	Threonine--tRNA ligase, cytoplasmic OS=Bos taurus GN=TARS PE=2 SV=1 - [SYTC_BOVIN]	CP	
Q3ZCH5	Zinc-alpha-2-glycoprotein OS=Bos taurus GN=AZGP1 PE=2 SV=1 - [ZA2G_BOVIN]	CP	S
Q3ZCI9	T-complex protein 1 subunit theta OS=Bos taurus GN=CCT8 PE=1 SV=3 - [TCPQ_BOVIN]	CP	S
Q4GZT4	ATP-binding cassette sub-family G member 2 OS=Bos taurus GN=ABCG2 PE=3 SV=2 - [ABCG2_BOVIN]	CP	S
Q58CW7	KIAA1068 protein OS=Bos taurus GN=KIAA1068 PE=2 SV=1 - [Q58CW7_BOVIN]	CP	
Q58DG0	Retinol binding protein 1, cellular OS=Bos taurus GN=RBP1 PE=2 SV=1 - [Q58DG0_BOVIN]	CP	S
Q5E9B5	Actin, gamma-enteric smooth muscle OS=Bos taurus GN=ACTG2 PE=2 SV=1 - [ACTH_BOVIN]	CP	S
Q5EA01	Beta-1,4-glucuronyltransferase 1 OS=Bos taurus GN=B4GAT1 PE=2 SV=2 - [B4GA1_BOVIN]	CP	
Q5MAB2	PLTP-like protein (Fragment) OS=Bos taurus PE=4 SV=1 - [Q5MAB2_BOVIN]		S
Q5USV7	Growth/differentiation factor 8 OS=Aepyceros melampus GN=MSTN PE=2 SV=1 - [GDF8_AEPME]		S
Q5YD57	Beta-casein (Fragment) OS=Capra hircus GN=CSN2 PE=4 SV=1 - [Q5YD57_CAPHI]	CP	S
Q69DJ1	Sulfate transporter OS=Bubalus bubalis GN=SLC26A2 PE=3 SV=1 - [S26A2_BUBBU]	CP	
Q69EZ6	CSCN1 OS=Capra hircus PE=2 SV=1 - [Q69EZ6_CAPHI]	CP	S
Q6LBN7	Lactoferrin (Fragment) OS=Bos taurus PE=2 SV=1 - [Q6LBN7_BOVIN]		S
Q6S4N9	Fatty acid binding protein 3 OS=Capra hircus GN=H-FABP PE=2 SV=1 - [Q6S4N9_CAPHI]	CP	S
Q6TMG6	Chitinase-3-like protein 1 OS=Ovis aries GN=CHI3L1 PE=1 SV=1 - [CH3L1_SHEEP]	CP	
Q6VUQ8	Beta-2-microglobulin (Fragment) OS=Bos taurus GN=B2M PE=4 SV=1 - [Q6VUQ8_BOVIN]		S

Q6XUZ5	Isocitrate dehydrogenase [NADP] cytoplasmic OS=Ovis aries GN=IDH1 PE=2 SV=1 - [IDHC_SHEEP]	CP	
Q6YLM4	Kappa casein (Fragment) OS=Cephalophus dorsalis PE=4 SV=1 - [Q6YLM4_CEPDO]	CP	S
Q71V69	Kappa-casein (Fragment) OS=Capra hircus PE=4 SV=1 - [Q71V69_CAPHI]	CP	
Q76LV1	Heat shock protein HSP 90-beta OS=Bos taurus GN=HSP90AB1 PE=2 SV=3 - [HS90B_BOVIN]	CP	
Q7M2Q9	Rho protein GDP-dissociation inhibitor (Fragments) OS=Bos taurus PE=1 SV=1 - [Q7M2Q9_BOVIN]	CP	
Q7M2U8	Apolipoprotein E OS=Ovis aries GN=APOE PE=2 SV=1 - [APOE_SHEEP]	CP	S
Q7M323	Plasminogen (Fragment) OS=Capra hircus GN=PLG PE=1 SV=1 - [PLMN_CAPHI]	CP	S
Q7M3G9	GTP-binding regulatory protein Gi alpha-2 chain (Fragments) OS=Bos taurus PE=4 SV=1 - [Q7M3G9_BOVIN]	CP	
Q861V5	Peptidyl-prolyl cis-trans isomerase (Fragment) OS=Bos taurus PE=2 SV=1 - [Q861V5_BOVIN]		S
Q862F6	Similar to elongation factor 1 alpha (Fragment) OS=Bos taurus PE=2 SV=1 - [Q862F6_BOVIN]		S
Q862J3	Similar to vacuolar H-ATPase subunit D (Fragment) OS=Bos taurus PE=2 SV=1 - [Q862J3_BOVIN]		S
Q862N7	Transcription factor BTF3 (Fragment) OS=Bos taurus PE=2 SV=1 - [Q862N7_BOVIN]	CP	
Q863B3	Staphylococcal nuclease domain-containing protein 1 OS=Bos taurus GN=SND1 PE=1 SV=1 - [SND1_BOVIN]	CP	S
Q8HZJ7	Heat shock protein-90 (Fragment) OS=Capra hircus PE=2 SV=1 - [Q8HZJ7_CAPHI]		S
Q8SPQ0	Chitinase-3-like protein 1 OS=Capra hircus GN=CHI3L1 PE=1 SV=1 - [CH3L1_CAPHI]		S
Q95L76	Beta-casein OS=Capra hircus GN=csn2 PE=3 SV=1 - [Q95L76_CAPHI]		S
Q95ME3	GTP binding protein Rab1a (Fragment) OS=Muntiacus reevesi PE=4 SV=1 - [Q95ME3_MUNRE]		S
Q95MX4	Kappa-casein (Fragment) OS=Oreotragus oreotragus PE=4 SV=1 - [Q95MX4_OREOR]	CP	S
Q95MX5	Kappa-casein (Fragment) OS=Neotragus moschatus PE=4 SV=1 - [Q95MX5_NEOMO]	CP	
Q95MY3	Kappa-casein (Fragment) OS=Redunca redunca PE=4 SV=1 - [Q95MY3_REDRE]	CP	
Q9BGL6	MMP-13 (Fragment) OS=Ovis aries PE=2 SV=1 - [Q9BGL6_SHEEP]	CP	S
Q9BGU5	Cathepsin D (Fragment) OS=Bos taurus GN=cat-D PE=2 SV=1 - [Q9BGU5_BOVIN]	CP	S
Q9GIJ9	MHC class II antigen (Fragment) OS=Damaliscus pygargus pygargus GN=DRB PE=4 SV=1 - [Q9GIJ9_DAMPY]	CP	S
Q9GK30	Parathyroid hormone-related protein (Fragment) OS=Ovis aries GN=PTHLH PE=2 SV=1 - [PTHR_SHEEP]	CP	
Q9MZY2	Airway lactoperoxidase OS=Ovis aries PE=1 SV=1 - [Q9MZY2_SHEEP]	CP	
Q9N109	Lactate dehydrogenase A (Fragment) OS=Ovis aries PE=2 SV=1 - [Q9N109_SHEEP]	CP	
Q9N258	Kappa-casein (Fragment) OS=Bos taurus x Bos indicus GN=CSN3 PE=4 SV=1 - [Q9N258_9CETA]	CP	
Q9N282	MMP-9 (Fragment) OS=Bos taurus GN=bmmp-9 PE=2 SV=1 - [Q9N282_BOVIN]	CP	
Q9TTP5	Pyridoxal kinase (Fragment) OS=Ovis aries PE=2 SV=1 - [Q9TTP5_SHEEP]	CP	
Q9TTR6	Putative sodium-glucose cotransporter (Fragment) OS=Bos taurus GN=sglt1 PE=2 SV=1 - [Q9TTR6_BOVIN]	CP	S
Q9TU03	Rho GDP-dissociation inhibitor 2 OS=Bos taurus GN=ARHGDIB PE=2 SV=3 - [GDIR2_BOVIN]	CP	S
Q9XSG3	Isocitrate dehydrogenase [NADP] cytoplasmic OS=Bos taurus GN=IDH1 PE=2 SV=1 - [IDHC_BOVIN]		S
Q9XSM0	Prostaglandin-H2 D-isomerase OS=Ovis aries GN=PTGDS PE=1 SV=1 - [PTGDS_SHEEP]	CP	
Q9XSQ8	MAP28 protein OS=Capra hircus GN=map28 PE=2 SV=1 - [Q9XSQ8_CAPHI]	CP	S
Q9XSQ9	Bac7.5 protein OS=Capra hircus GN=bac7.5 PE=2 SV=1 - [Q9XSQ9_CAPHI]	CP	S
Q9XSY9	Osteopontin OS=Ovis aries GN=SPP1 PE=2 SV=1 - [OSTP_SHEEP]	CP	
Q9XT27	Ceruloplasmin OS=Ovis aries GN=CP PE=2 SV=1 - [CERU_SHEEP]		S
R4ZCQ9	Ubiquitin (Fragment) OS=Bubalus bubalis GN=UBI PE=2 SV=1 - [R4ZCQ9_BUBBU]		S
S5DFV7	Ribonuclease III (Fragment) OS=Bubalus bubalis GN=Dicer1 PE=2 SV=1 - [S5DFV7_BUBBU]		S
S5FR89	Cathepsin B OS=Ovis aries GN=CTSB PE=2 SV=1 - [S5FR89_SHEEP]	CP	S
S5G3C2	Insulin-like growth factor 2 receptor (Fragment) OS=Bubalus bubalis GN=IGF2R PE=2 SV=1 - [S5G3C2_BUBBU]	CP	
U5Y4C2	Beta-casein (Fragment) OS=Bos indicus PE=4 SV=1 - [U5Y4C2_BOSIN]	CP	
V5RKP7	Beta lactoglobulin (Fragment) OS=Bos indicus GN=LGB PE=4 SV=1 - [V5RKP7_BOSIN]	CP	S
V9XY47	Alpha-mannosidase OS=Capra hircus PE=2 SV=1 - [V9XY47_CAPHI]	CP	
W5NPK5	Uncharacterized protein OS=Ovis aries GN=C3 PE=4 SV=1 - [W5NPK5_SHEEP]	CP	S
W5NPW2	Uncharacterized protein OS=Ovis aries PE=4 SV=1 - [W5NPW2_SHEEP]	CP	
W5NQ46	Fibrinogen beta chain OS=Ovis aries GN=FGB PE=4 SV=1 - [W5NQ46_SHEEP]	CP	S
W5NQH6	Protein S100 OS=Ovis aries GN=S100A9 PE=3 SV=1 - [W5NQH6_SHEEP]	CP	
W5NQK9	Uncharacterized protein (Fragment) OS=Ovis aries GN=S100A8 PE=4 SV=1 - [W5NQK9_SHEEP]	CP	
W5NQM4	Uncharacterized protein (Fragment) OS=Ovis aries GN=IL20RA PE=4 SV=1 - [W5NQM4_SHEEP]	CP	
W5NQS8	Uncharacterized protein (Fragment) OS=Ovis aries GN=LRFN2 PE=4 SV=1 - [W5NQS8_SHEEP]		S
W5NQW9	Uncharacterized protein OS=Ovis aries GN=LOC101104482 PE=4 SV=1 - [W5NQW9_SHEEP]	CP	S
W5NRG7	Uncharacterized protein OS=Ovis aries GN=ITIH4 PE=4 SV=1 - [W5NRG7_SHEEP]	CP	S
W5NRH2	Uncharacterized protein OS=Ovis aries GN=LOC101103133 PE=4 SV=1 - [W5NRH2_SHEEP]	CP	S
W5NRI1	Uncharacterized protein OS=Ovis aries GN=LOC101113831 PE=4 SV=1 - [W5NRI1_SHEEP]	CP	S
W5NRR2	Uncharacterized protein OS=Ovis aries GN=FAM35A PE=4 SV=1 - [W5NRR2_SHEEP]	CP	
W5NSA6	Uncharacterized protein OS=Ovis aries GN=A2M PE=4 SV=1 - [W5NSA6_SHEEP]	CP	S
W5NSN2	Uncharacterized protein OS=Ovis aries GN=COL16A1 PE=4 SV=1 - [W5NSN2_SHEEP]	CP	
W5NSV0	Uncharacterized protein OS=Ovis aries GN=LTBP2 PE=4 SV=1 - [W5NSV0_SHEEP]		S
W5NT24	Uncharacterized protein OS=Ovis aries GN=TNXB PE=4 SV=1 - [W5NT24_SHEEP]		S
W5NTD7	Uncharacterized protein OS=Ovis aries GN=WDR49 PE=4 SV=1 - [W5NTD7_SHEEP]	CP	
W5NTG6	Uncharacterized protein (Fragment) OS=Ovis aries GN=TINAGL1 PE=3 SV=1 - [W5NTG6_SHEEP]	CP	
W5NTW3	Uncharacterized protein (Fragment) OS=Ovis aries GN=ITIH1 PE=4 SV=1 - [W5NTW3_SHEEP]	CP	S
W5NUV2	Uncharacterized protein (Fragment) OS=Ovis aries GN=ARF5 PE=3 SV=1 - [W5NUV2_SHEEP]	CP	
W5NUX8	Uncharacterized protein OS=Ovis aries PE=4 SV=1 - [W5NUX8_SHEEP]	CP	S
W5NVB2	Uncharacterized protein OS=Ovis aries PE=3 SV=1 - [W5NVB2_SHEEP]		S
W5NVE9	Uncharacterized protein OS=Ovis aries GN=NADK PE=3 SV=1 - [W5NVE9_SHEEP]		S
W5NVI9	Uncharacterized protein (Fragment) OS=Ovis aries GN=MED17 PE=4 SV=1 - [W5NVI9_SHEEP]	CP	
W5NVV6	Uncharacterized protein (Fragment) OS=Ovis aries GN=DNAJC3 PE=4 SV=1 - [W5NVV6_SHEEP]	CP	
W5NW80	Uncharacterized protein (Fragment) OS=Ovis aries GN=GAA PE=3 SV=1 - [W5NW80_SHEEP]	CP	
W5NW93	Uncharacterized protein OS=Ovis aries GN=KMT2E PE=4 SV=1 - [W5NW93_SHEEP]		S
W5NXA9	Uncharacterized protein (Fragment) OS=Ovis aries GN=LENG9 PE=4 SV=1 - [W5NXA9_SHEEP]	CP	
W5NXP3	Uncharacterized protein OS=Ovis aries GN=LOC101111083 PE=3 SV=1 - [W5NXP3_SHEEP]	CP	S
W5NXW9	Uncharacterized protein OS=Ovis aries GN=IGHM PE=4 SV=1 - [W5NXW9_SHEEP]	CP	S
W5NXZ5	Uncharacterized protein (Fragment) OS=Ovis aries GN=FHOD1 PE=4 SV=1 - [W5NXZ5_SHEEP]	CP	
W5NYZ7	Uncharacterized protein OS=Ovis aries GN=CREB3L1 PE=4 SV=1 - [W5NYZ7_SHEEP]	CP	S
W5NZ31	Uncharacterized protein (Fragment) OS=Ovis aries GN=AHCTF1 PE=4 SV=1 - [W5NZ31_SHEEP]	CP	
W5NZ32	Uncharacterized protein OS=Ovis aries GN=CANT1 PE=4 SV=1 - [W5NZ32_SHEEP]		S
W5NZ59	Uncharacterized protein OS=Ovis aries GN=CCDC136 PE=4 SV=1 - [W5NZ59_SHEEP]		S
W5NZG6	Uncharacterized protein (Fragment) OS=Ovis aries GN=GBX1 PE=4 SV=1 - [W5NZG6_SHEEP]	CP	
W5NZU3	Uncharacterized protein OS=Ovis aries GN=CDH1 PE=4 SV=1 - [W5NZU3_SHEEP]	CP	S
W5NZY4	Uncharacterized protein OS=Ovis aries GN=LOC101110632 PE=4 SV=1 - [W5NZY4_SHEEP]	CP	
W5P0K6	Uncharacterized protein OS=Ovis aries GN=PMFBP1 PE=4 SV=1 - [W5P0K6_SHEEP]		S
W5P0P5	Uncharacterized protein (Fragment) OS=Ovis aries GN=CACNA1G PE=4 SV=1 - [W5P0P5_SHEEP]		S
W5P0Q7	Uncharacterized protein OS=Ovis aries GN=TMLHE PE=4 SV=1 - [W5P0Q7_SHEEP]	CP	
W5P0U4	Uncharacterized protein OS=Ovis aries GN=MUC1 PE=4 SV=1 - [W5P0U4_SHEEP]	CP	
W5P0V6	Uncharacterized protein OS=Ovis aries GN=SCCPDH PE=4 SV=1 - [W5P0V6_SHEEP]	CP	

W5P0W4	Uncharacterized protein (Fragment) OS=Ovis aries GN=SEMA7A PE=4 SV=1 - [W5P0W4_SHEEP]	CP	S
W5P0Y1	Uncharacterized protein (Fragment) OS=Ovis aries GN=RYR2 PE=4 SV=1 - [W5P0Y1_SHEEP]	CP	
W5P182	Uncharacterized protein OS=Ovis aries GN=RPS7 PE=4 SV=1 - [W5P182_SHEEP]	CP	
W5P1C2	Uncharacterized protein OS=Ovis aries GN=NUCB2 PE=4 SV=1 - [W5P1C2_SHEEP]	CP	S
W5P285	Glucose-6-phosphate 1-dehydrogenase OS=Ovis aries PE=3 SV=1 - [W5P285_SHEEP]		S
W5P2R0	Uncharacterized protein (Fragment) OS=Ovis aries PE=4 SV=1 - [W5P2R0_SHEEP]	CP	
W5P2V0	Uncharacterized protein (Fragment) OS=Ovis aries GN=EZR PE=4 SV=1 - [W5P2V0_SHEEP]	CP	
W5P2V3	Uncharacterized protein OS=Ovis aries GN=PEPD PE=3 SV=1 - [W5P2V3_SHEEP]	CP	S
W5P323	Glucose-6-phosphate isomerase OS=Ovis aries GN=GPI PE=3 SV=1 - [W5P323_SHEEP]	CP	
W5P3A1	Uncharacterized protein (Fragment) OS=Ovis aries PE=4 SV=1 - [W5P3A1_SHEEP]		S
W5P3C6	Beta-hexosaminidase OS=Ovis aries GN=HEXB PE=3 SV=1 - [W5P3C6_SHEEP]	CP	
W5P3H0	Uncharacterized protein OS=Ovis aries GN=ZNF385D PE=4 SV=1 - [W5P3H0_SHEEP]	CP	S
W5P3H8	Uncharacterized protein OS=Ovis aries GN=IGF2R PE=4 SV=1 - [W5P3H8_SHEEP]		S
W5P3L1	Sodium channel protein OS=Ovis aries GN=SCN9A PE=3 SV=1 - [W5P3L1_SHEEP]	CP	
W5P3X0	Uncharacterized protein OS=Ovis aries GN=NACA2 PE=4 SV=1 - [W5P3X0_SHEEP]	CP	S
W5P4S5	Ceruloplasmin OS=Ovis aries GN=CP PE=4 SV=1 - [W5P4S5_SHEEP]	CP	
W5P550	Uncharacterized protein OS=Ovis aries GN=DNAH1 PE=4 SV=1 - [W5P550_SHEEP]	CP	
W5P5G3	Uncharacterized protein (Fragment) OS=Ovis aries GN=PXDN PE=4 SV=1 - [W5P5G3_SHEEP]		S
W5P5I0	Uncharacterized protein OS=Ovis aries GN=CFI PE=3 SV=1 - [W5P5I0_SHEEP]	CP	S
W5P5R4	Uncharacterized protein (Fragment) OS=Ovis aries PE=4 SV=1 - [W5P5R4_SHEEP]	CP	
W5P5R6	Uncharacterized protein (Fragment) OS=Ovis aries GN=SPIN4 PE=4 SV=1 - [W5P5R6_SHEEP]	CP	
W5P5W9	Triosephosphate isomerase (Fragment) OS=Ovis aries GN=TPI1 PE=3 SV=1 - [W5P5W9_SHEEP]	CP	S
W5P601	Uncharacterized protein OS=Ovis aries GN=STOM PE=4 SV=1 - [W5P601_SHEEP]		S
W5P648	Uncharacterized protein OS=Ovis aries GN=RAB14 PE=3 SV=1 - [W5P648_SHEEP]	CP	S
W5P6E0	Uncharacterized protein OS=Ovis aries GN=OS9 PE=4 SV=1 - [W5P6E0_SHEEP]	CP	S
W5P6F4	Uncharacterized protein OS=Ovis aries GN=C5 PE=4 SV=1 - [W5P6F4_SHEEP]	CP	
W5P6F9	Uncharacterized protein OS=Ovis aries GN=DPP3 PE=4 SV=1 - [W5P6F9_SHEEP]	CP	
W5P6J0	Uncharacterized protein OS=Ovis aries GN=PHF19 PE=4 SV=1 - [W5P6J0_SHEEP]	CP	
W5P6V4	Uncharacterized protein OS=Ovis aries GN=GLG1 PE=4 SV=1 - [W5P6V4_SHEEP]		S
W5P708	Uncharacterized protein OS=Ovis aries GN=ACTN4 PE=4 SV=1 - [W5P708_SHEEP]	CP	S
W5P7H6	Uncharacterized protein (Fragment) OS=Ovis aries PE=4 SV=1 - [W5P7H6_SHEEP]	CP	
W5P7W2	Uncharacterized protein (Fragment) OS=Ovis aries GN=SDF4 PE=4 SV=1 - [W5P7W2_SHEEP]	CP	S
W5P8E9	Uncharacterized protein (Fragment) OS=Ovis aries GN=CD109 PE=4 SV=1 - [W5P8E9_SHEEP]	CP	S
W5P8F9	Uncharacterized protein OS=Ovis aries PE=4 SV=1 - [W5P8F9_SHEEP]	CP	S
W5P8W2	Uncharacterized protein OS=Ovis aries GN=MGAT5B PE=4 SV=1 - [W5P8W2_SHEEP]	CP	
W5P9K8	Uncharacterized protein OS=Ovis aries GN=PTH1R PE=4 SV=1 - [W5P9K8_SHEEP]		S
W5P9M2	Uncharacterized protein OS=Ovis aries GN=PTGR1 PE=4 SV=1 - [W5P9M2_SHEEP]	CP	
W5P9S1	Uncharacterized protein OS=Ovis aries GN=SPTBN4 PE=4 SV=1 - [W5P9S1_SHEEP]		S
W5P9U5	Uncharacterized protein OS=Ovis aries GN=LOC101104114 PE=3 SV=1 - [W5P9U5_SHEEP]	CP	S
W5P9V5	Uncharacterized protein OS=Ovis aries PE=4 SV=1 - [W5P9V5_SHEEP]	CP	S
W5PA72	Uncharacterized protein OS=Ovis aries PE=4 SV=1 - [W5PA72_SHEEP]	CP	
W5PA89	Uncharacterized protein OS=Ovis aries GN=ST6GALNAC2 PE=4 SV=1 - [W5PA89_SHEEP]		S
W5PAH7	Uncharacterized protein OS=Ovis aries GN=KBTBD6 PE=4 SV=1 - [W5PAH7_SHEEP]	CP	
W5PAJ2	Uncharacterized protein OS=Ovis aries GN=PSAP PE=4 SV=1 - [W5PAJ2_SHEEP]	CP	S
W5PAJ3	Uncharacterized protein OS=Ovis aries GN=BPIFB1 PE=4 SV=1 - [W5PAJ3_SHEEP]	CP	
W5PAM0	Uncharacterized protein OS=Ovis aries GN=SETD5 PE=4 SV=1 - [W5PAM0_SHEEP]	CP	
W5PAQ4	Uncharacterized protein (Fragment) OS=Ovis aries GN=FUCA1 PE=4 SV=1 - [W5PAQ4_SHEEP]	CP	S
W5PAT6	Uncharacterized protein (Fragment) OS=Ovis aries GN=LOC101113832 PE=4 SV=1 - [W5PAT6_SHEEP]	CP	S
W5PB98	Uncharacterized protein (Fragment) OS=Ovis aries GN=TPRA1 PE=4 SV=1 - [W5PB98_SHEEP]	CP	
W5PBF4	Uncharacterized protein OS=Ovis aries GN=ZNF521 PE=4 SV=1 - [W5PBF4_SHEEP]		S
W5PBW1	Uncharacterized protein (Fragment) OS=Ovis aries GN=SDS PE=4 SV=1 - [W5PBW1_SHEEP]	CP	S
W5PBY2	Uncharacterized protein OS=Ovis aries GN=C4BPA PE=4 SV=1 - [W5PBY2_SHEEP]	CP	
W5PCE0	Uncharacterized protein OS=Ovis aries GN=PLBD2 PE=4 SV=1 - [W5PCE0_SHEEP]	CP	S
W5PCT4	Uncharacterized protein OS=Ovis aries GN=SLC34A2 PE=4 SV=1 - [W5PCT4_SHEEP]	CP	S
W5PD86	Uncharacterized protein OS=Ovis aries GN=POLH PE=4 SV=1 - [W5PD86_SHEEP]	CP	
W5PDI8	Uncharacterized protein OS=Ovis aries GN=GSDMC PE=4 SV=1 - [W5PDI8_SHEEP]	CP	
W5PDR7	Uncharacterized protein OS=Ovis aries GN=C8A PE=4 SV=1 - [W5PDR7_SHEEP]		S
W5PDY2	Uncharacterized protein OS=Ovis aries PE=4 SV=1 - [W5PDY2_SHEEP]		S
W5PE22	Uncharacterized protein (Fragment) OS=Ovis aries GN=GDI2 PE=4 SV=1 - [W5PE22_SHEEP]	CP	S
W5PE31	Uncharacterized protein (Fragment) OS=Ovis aries GN=FUT11 PE=3 SV=1 - [W5PE31_SHEEP]	CP	S
W5PEL7	Uncharacterized protein (Fragment) OS=Ovis aries GN=HSPG2 PE=4 SV=1 - [W5PEL7_SHEEP]		S
W5PEY4	Uncharacterized protein OS=Ovis aries GN=TCN2 PE=4 SV=1 - [W5PEY4_SHEEP]	CP	S
W5PFB9	Alkaline phosphatase OS=Ovis aries GN=ALPL PE=3 SV=1 - [W5PFB9_SHEEP]	CP	
W5PFC9	Uncharacterized protein (Fragment) OS=Ovis aries GN=LOC101117129 PE=4 SV=1 - [W5PFC9_SHEEP]	CP	S
W5PFF9	Uncharacterized protein OS=Ovis aries PE=4 SV=1 - [W5PFF9_SHEEP]	CP	S
W5PFJ0	Uncharacterized protein OS=Ovis aries GN=VCL PE=4 SV=1 - [W5PFJ0_SHEEP]	CP	S
W5PFM6	Uncharacterized protein OS=Ovis aries GN=IQCD PE=4 SV=1 - [W5PFM6_SHEEP]		S
W5PFS5	Uncharacterized protein (Fragment) OS=Ovis aries PE=4 SV=1 - [W5PFS5_SHEEP]		S
W5PFV6	Uncharacterized protein (Fragment) OS=Ovis aries GN=ZNF341 PE=4 SV=1 - [W5PFV6_SHEEP]		S
W5PFW2	Uncharacterized protein OS=Ovis aries GN=NEB PE=4 SV=1 - [W5PFW2_SHEEP]		S
W5PG41	Uncharacterized protein OS=Ovis aries GN=H6PD PE=4 SV=1 - [W5PG41_SHEEP]	CP	
W5PG90	Uncharacterized protein OS=Ovis aries GN=PRSS8 PE=3 SV=1 - [W5PG90_SHEEP]	CP	S
W5PGE9	Uncharacterized protein (Fragment) OS=Ovis aries PE=4 SV=1 - [W5PGE9_SHEEP]	CP	S
W5PGL5	Sodium/nucleoside cotransporter (Fragment) OS=Ovis aries GN=SLC28A3 PE=3 SV=1 - [W5PGL5_SHEEP]	CP	S
W5PGL9	Elongation factor 1-alpha OS=Ovis aries PE=3 SV=1 - [W5PGL9_SHEEP]	CP	
W5PGP1	Uncharacterized protein OS=Ovis aries GN=LPO PE=4 SV=1 - [W5PGP1_SHEEP]		S
W5PGT9	Uncharacterized protein OS=Ovis aries GN=IGHE PE=4 SV=1 - [W5PGT9_SHEEP]	CP	S
W5PH81	Uncharacterized protein OS=Ovis aries GN=C7 PE=4 SV=1 - [W5PH81_SHEEP]	CP	S
W5PH95	Uncharacterized protein (Fragment) OS=Ovis aries PE=4 SV=1 - [W5PH95_SHEEP]	CP	S
W5PHI7	Uncharacterized protein OS=Ovis aries GN=LOC101116892 PE=3 SV=1 - [W5PHI7_SHEEP]	CP	
W5PHJ1	Uncharacterized protein (Fragment) OS=Ovis aries GN=CEACAM16 PE=4 SV=1 - [W5PHJ1_SHEEP]		S
W5PHP7	Uncharacterized protein OS=Ovis aries GN=LOC101117146 PE=3 SV=1 - [W5PHP7_SHEEP]	CP	S
W5PHP8	Uncharacterized protein (Fragment) OS=Ovis aries GN=LRG1 PE=4 SV=1 - [W5PHP8_SHEEP]	CP	S
W5PHS2	Uncharacterized protein OS=Ovis aries GN=LOC101116799 PE=3 SV=1 - [W5PHS2_SHEEP]	CP	S
W5PI67	Uncharacterized protein OS=Ovis aries GN=IDS PE=4 SV=1 - [W5PI67_SHEEP]	CP	S
W5PID9	Uncharacterized protein OS=Ovis aries GN=C9 PE=4 SV=1 - [W5PID9_SHEEP]	CP	S
W5PIW6	Uncharacterized protein OS=Ovis aries GN=PRTN3 PE=3 SV=1 - [W5PIW6_SHEEP]	CP	

W5PJ93	Uncharacterized protein OS=Ovis aries GN=RNF220 PE=4 SV=1 - [W5PJ93_SHEEP]		S
W5PJI1	Uncharacterized protein OS=Ovis aries GN=IGSF5 PE=4 SV=1 - [W5PJI1_SHEEP]	CP	
W5PJJ7	Uncharacterized protein (Fragment) OS=Ovis aries GN=LOC101109421 PE=4 SV=1 - [W5PJJ7_SHEEP]	CP	
W5PJN2	Uncharacterized protein OS=Ovis aries PE=4 SV=1 - [W5PJN2_SHEEP]	CP	
W5PJP9	Uncharacterized protein OS=Ovis aries GN=SYNGAP1 PE=4 SV=1 - [W5PJP9_SHEEP]	CP	S
W5PJZ2	Uncharacterized protein OS=Ovis aries GN=SERPING1 PE=3 SV=1 - [W5PJZ2_SHEEP]	CP	
W5PJZ9	Uncharacterized protein OS=Ovis aries GN=STC1 PE=4 SV=1 - [W5PJZ9_SHEEP]	CP	
W5PK41	Uncharacterized protein OS=Ovis aries GN=LPIN2 PE=4 SV=1 - [W5PK41_SHEEP]		S
W5PK51	Uncharacterized protein OS=Ovis aries GN=STAP2 PE=4 SV=1 - [W5PK51_SHEEP]		S
W5PLB7	Peptidoglycan-recognition protein OS=Ovis aries GN=PGLYRP1 PE=3 SV=1 - [W5PLB7_SHEEP]	CP	S
W5PM49	Uncharacterized protein OS=Ovis aries GN=USH2A PE=4 SV=1 - [W5PM49_SHEEP]	CP	S
W5PM94	Uncharacterized protein OS=Ovis aries GN=ACSS2 PE=4 SV=1 - [W5PM94_SHEEP]	CP	
W5PMH6	Uncharacterized protein (Fragment) OS=Ovis aries GN=LCN2 PE=3 SV=1 - [W5PMH6_SHEEP]	CP	S
W5PMM7	Protein disulfide-isomerase (Fragment) OS=Ovis aries GN=PDIA3 PE=3 SV=1 - [W5PMM7_SHEEP]	CP	S
W5PMT0	Uncharacterized protein OS=Ovis aries GN=XDH PE=4 SV=1 - [W5PMT0_SHEEP]	CP	S
W5PN01	Uncharacterized protein OS=Ovis aries GN=NDUFV3 PE=4 SV=1 - [W5PN01_SHEEP]	CP	
W5PN09	Uncharacterized protein (Fragment) OS=Ovis aries PE=4 SV=1 - [W5PN09_SHEEP]		S
W5PNP1	Uncharacterized protein OS=Ovis aries GN=MFGE8 PE=4 SV=1 - [W5PNP1_SHEEP]	CP	
W5PNQ0	Uncharacterized protein OS=Ovis aries GN=ZFYVE27 PE=4 SV=1 - [W5PNQ0_SHEEP]	CP	
W5PP27	Uncharacterized protein (Fragment) OS=Ovis aries GN=LOC101113257 PE=4 SV=1 - [W5PP27_SHEEP]	CP	
W5PPS0	Uncharacterized protein OS=Ovis aries GN=LOC101112330 PE=4 SV=1 - [W5PPS0_SHEEP]	CP	
W5PQI5	Uncharacterized protein OS=Ovis aries GN=METTL11B PE=4 SV=1 - [W5PQI5_SHEEP]	CP	
W5PRU0	Uncharacterized protein OS=Ovis aries GN=RHAG PE=4 SV=1 - [W5PRU0_SHEEP]	CP	
W5PS71	Uncharacterized protein OS=Ovis aries GN=CASP8AP2 PE=4 SV=1 - [W5PS71_SHEEP]	CP	S
W5PS86	Uncharacterized protein OS=Ovis aries GN=ESPN PE=4 SV=1 - [W5PS86_SHEEP]		S
W5PS94	Uncharacterized protein OS=Ovis aries GN=NUCB1 PE=4 SV=1 - [W5PS94_SHEEP]	CP	S
W5PSA3	Uncharacterized protein OS=Ovis aries GN=TCN1 PE=4 SV=1 - [W5PSA3_SHEEP]		S
W5PSJ8	Uncharacterized protein (Fragment) OS=Ovis aries PE=4 SV=1 - [W5PSJ8_SHEEP]		S
W5PSK1	Uncharacterized protein OS=Ovis aries GN=HYOU1 PE=3 SV=1 - [W5PSK1_SHEEP]		S
W5PSQ7	Uncharacterized protein OS=Ovis aries PE=4 SV=1 - [W5PSQ7_SHEEP]	CP	S
W5PSZ8	Uncharacterized protein (Fragment) OS=Ovis aries GN=DAK PE=4 SV=1 - [W5PSZ8_SHEEP]		S
W5PTG9	Uncharacterized protein OS=Ovis aries GN=GC PE=4 SV=1 - [W5PTG9_SHEEP]	CP	S
W5PTS8	Uncharacterized protein OS=Ovis aries GN=MLEC PE=4 SV=1 - [W5PTS8_SHEEP]	CP	
W5PU70	Uncharacterized protein OS=Ovis aries GN=MUC16 PE=4 SV=1 - [W5PU70_SHEEP]	CP	S
W5PUH7	Uncharacterized protein (Fragment) OS=Ovis aries GN=GSTP1 PE=3 SV=1 - [W5PUH7_SHEEP]	CP	S
W5PUV3	Uncharacterized protein OS=Ovis aries GN=NT5E PE=3 SV=1 - [W5PUV3_SHEEP]		S
W5PV11	Uncharacterized protein OS=Ovis aries GN=IRF3 PE=4 SV=1 - [W5PV11_SHEEP]	CP	
W5PV27	Uncharacterized protein OS=Ovis aries PE=4 SV=1 - [W5PV27_SHEEP]		S
W5PV41	Uncharacterized protein OS=Ovis aries GN=HHIPL2 PE=4 SV=1 - [W5PV41_SHEEP]	CP	S
W5PV84	Amino acid transporter (Fragment) OS=Ovis aries GN=EAAT3 PE=3 SV=1 - [W5PV84_SHEEP]	CP	S
W5PVJ8	Uncharacterized protein OS=Ovis aries PE=4 SV=1 - [W5PVJ8_SHEEP]	CP	
W5PVQ2	Protein-tyrosine-phosphatase OS=Ovis aries GN=PTPRK PE=4 SV=1 - [W5PVQ2_SHEEP]		S
W5PVT4	Uncharacterized protein OS=Ovis aries GN=GALNS PE=4 SV=1 - [W5PVT4_SHEEP]	CP	
W5PVV9	Uncharacterized protein (Fragment) OS=Ovis aries GN=MIA3 PE=4 SV=1 - [W5PVV9_SHEEP]	CP	
W5PVZ2	Uncharacterized protein OS=Ovis aries GN=RB1CC1 PE=4 SV=1 - [W5PVZ2_SHEEP]		S
W5PVZ9	Uncharacterized protein OS=Ovis aries GN=DAG1 PE=4 SV=1 - [W5PVZ9_SHEEP]	CP	
W5PW21	Uncharacterized protein OS=Ovis aries GN=ITIH2 PE=4 SV=1 - [W5PW21_SHEEP]	CP	S
W5PWM0	Uncharacterized protein OS=Ovis aries PE=4 SV=1 - [W5PWM0_SHEEP]	CP	
W5PXC8	Uncharacterized protein OS=Ovis aries GN=SERPINF2 PE=3 SV=1 - [W5PXC8_SHEEP]	CP	
W5PXF5	Uncharacterized protein (Fragment) OS=Ovis aries GN=PROM2 PE=4 SV=1 - [W5PXF5_SHEEP]	CP	S
W5PXP4	Uncharacterized protein OS=Ovis aries GN=ABCA10 PE=3 SV=1 - [W5PXP4_SHEEP]	CP	
W5PXY0	Uncharacterized protein OS=Ovis aries GN=SYDE2 PE=4 SV=1 - [W5PXY0_SHEEP]	CP	
W5PY68	Lipase OS=Ovis aries GN=LIPK PE=3 SV=1 - [W5PY68_SHEEP]	CP	
W5PYG2	Uncharacterized protein OS=Ovis aries GN=VNN1 PE=4 SV=1 - [W5PYG2_SHEEP]	CP	S
W5PYV5	Uncharacterized protein OS=Ovis aries GN=FASLG PE=4 SV=1 - [W5PYV5_SHEEP]	CP	
W5PYW3	C-X-C motif chemokine OS=Ovis aries GN=LOC101103238 PE=3 SV=1 - [W5PYW3_SHEEP]	CP	S
W5PZ18	Uncharacterized protein OS=Ovis aries GN=SMARCA1 PE=4 SV=1 - [W5PZ18_SHEEP]	CP	
W5PZ65	Uncharacterized protein (Fragment) OS=Ovis aries GN=PSMA7 PE=4 SV=1 - [W5PZ65_SHEEP]		S
W5PZ94	Aconitate hydratase OS=Ovis aries GN=ACO1 PE=3 SV=1 - [W5PZ94_SHEEP]		S
W5PZC6	Uncharacterized protein (Fragment) OS=Ovis aries GN=SPIRE2 PE=4 SV=1 - [W5PZC6_SHEEP]	CP	
W5PZI1	Clusterin OS=Ovis aries GN=CLU PE=3 SV=1 - [W5PZI1_SHEEP]	CP	S
W5PZU1	Uncharacterized protein OS=Ovis aries PE=4 SV=1 - [W5PZU1_SHEEP]		S
W5PZX0	Uncharacterized protein OS=Ovis aries GN=KRT24 PE=3 SV=1 - [W5PZX0_SHEEP]	CP	S
W5Q021	Uncharacterized protein (Fragment) OS=Ovis aries GN=FAM20A PE=4 SV=1 - [W5Q021_SHEEP]	CP	S
W5Q051	Uncharacterized protein OS=Ovis aries GN=ZNF432 PE=4 SV=1 - [W5Q051_SHEEP]		S
W5Q076	Uncharacterized protein (Fragment) OS=Ovis aries GN=B3GNT3 PE=4 SV=1 - [W5Q076_SHEEP]		S
W5Q0F3	Uncharacterized protein (Fragment) OS=Ovis aries GN=TGFBI PE=4 SV=1 - [W5Q0F3_SHEEP]	CP	
W5Q0V2	Uncharacterized protein (Fragment) OS=Ovis aries GN=BTD PE=4 SV=1 - [W5Q0V2_SHEEP]	CP	S
W5Q0X5	Uncharacterized protein OS=Ovis aries GN=LOC101115576 PE=3 SV=1 - [W5Q0X5_SHEEP]	CP	
W5Q124	Uncharacterized protein OS=Ovis aries GN=LOC101119509 PE=3 SV=1 - [W5Q124_SHEEP]	CP	S
W5Q1E9	Uncharacterized protein OS=Ovis aries GN=DIXDC1 PE=4 SV=1 - [W5Q1E9_SHEEP]		S
W5Q1W2	Uncharacterized protein OS=Ovis aries GN=SDCBP PE=4 SV=1 - [W5Q1W2_SHEEP]	CP	
W5Q2T8	Uncharacterized protein (Fragment) OS=Ovis aries GN=METRNL PE=4 SV=1 - [W5Q2T8_SHEEP]	CP	
W5Q302	Uncharacterized protein OS=Ovis aries GN=GANAB PE=3 SV=1 - [W5Q302_SHEEP]	CP	
W5Q305	Uncharacterized protein (Fragment) OS=Ovis aries GN=GANAB PE=3 SV=1 - [W5Q305_SHEEP]		S
W5Q306	Uncharacterized protein OS=Ovis aries GN=ICAM3 PE=4 SV=1 - [W5Q306_SHEEP]		S
W5Q329	Uncharacterized protein OS=Ovis aries GN=UGGT1 PE=4 SV=1 - [W5Q329_SHEEP]	CP	S
W5Q3A3	Uncharacterized protein (Fragment) OS=Ovis aries GN=STK32C PE=4 SV=1 - [W5Q3A3_SHEEP]	CP	
W5Q3K5	Uncharacterized protein (Fragment) OS=Ovis aries PE=4 SV=1 - [W5Q3K5_SHEEP]	CP	
W5Q3Q3	Uncharacterized protein OS=Ovis aries GN=CPE PE=4 SV=1 - [W5Q3Q3_SHEEP]	CP	
W5Q411	Uncharacterized protein OS=Ovis aries GN=HSPA13 PE=3 SV=1 - [W5Q411_SHEEP]		S
W5Q430	Uncharacterized protein (Fragment) OS=Ovis aries GN=IDUA PE=4 SV=1 - [W5Q430_SHEEP]		S
W5Q4H0	Uncharacterized protein (Fragment) OS=Ovis aries GN=TERT PE=4 SV=1 - [W5Q4H0_SHEEP]	CP	
W5Q532	Uncharacterized protein (Fragment) OS=Ovis aries GN=PRDX4 PE=4 SV=1 - [W5Q532_SHEEP]	CP	S
W5Q5A6	Uncharacterized protein OS=Ovis aries GN=FGG PE=4 SV=1 - [W5Q5A6_SHEEP]	CP	S
W5Q5G8	Transketolase OS=Ovis aries GN=TKT PE=3 SV=1 - [W5Q5G8_SHEEP]	CP	S
W5Q5H8	Fibrinogen alpha chain OS=Ovis aries GN=FGA PE=4 SV=1 - [W5Q5H8_SHEEP]	CP	S

W5Q5L3	Uncharacterized protein OS=Ovis aries GN=DNAH14 PE=4 SV=1 - [W5Q5L3_SHEEP]	CP	
W5Q5W1	Uncharacterized protein OS=Ovis aries GN=SIL1 PE=4 SV=1 - [W5Q5W1_SHEEP]	CP	S
W5Q5Z3	Uncharacterized protein OS=Ovis aries GN=KRT77 PE=3 SV=1 - [W5Q5Z3_SHEEP]		S
W5Q689	Uncharacterized protein OS=Ovis aries GN=TPP1 PE=4 SV=1 - [W5Q689_SHEEP]	CP	
W5Q695	Transporter OS=Ovis aries GN=SLC6A14 PE=3 SV=1 - [W5Q695_SHEEP]		S
W5Q6E8	Uncharacterized protein OS=Ovis aries GN=KRT75 PE=3 SV=1 - [W5Q6E8_SHEEP]		S
W5Q6I8	Uncharacterized protein OS=Ovis aries GN=MTFR1 PE=4 SV=1 - [W5Q6I8_SHEEP]	CP	
W5Q6N8	Uncharacterized protein OS=Ovis aries GN=ABCA4 PE=4 SV=1 - [W5Q6N8_SHEEP]	CP	
W5Q6P7	Uncharacterized protein OS=Ovis aries GN=C3orf38 PE=4 SV=1 - [W5Q6P7_SHEEP]	CP	S
W5Q6Q0	Uncharacterized protein (Fragment) OS=Ovis aries GN=SCAMP1 PE=4 SV=1 - [W5Q6Q0_SHEEP]	CP	
W5Q750	Uncharacterized protein OS=Ovis aries GN=PROS1 PE=4 SV=1 - [W5Q750_SHEEP]		S
W5Q7I2	Uncharacterized protein OS=Ovis aries PE=4 SV=1 - [W5Q7I2_SHEEP]	CP	S
W5Q8P9	Uncharacterized protein (Fragment) OS=Ovis aries GN=IRF2BP2 PE=4 SV=1 - [W5Q8P9_SHEEP]		S
W5Q8S6	Uncharacterized protein OS=Ovis aries GN=CTCFL PE=4 SV=1 - [W5Q8S6_SHEEP]		S
W5Q989	Uncharacterized protein (Fragment) OS=Ovis aries GN=PLOD1 PE=4 SV=1 - [W5Q989_SHEEP]	CP	S
W5Q9A2	Uncharacterized protein (Fragment) OS=Ovis aries GN=AZGP1 PE=3 SV=1 - [W5Q9A2_SHEEP]	CP	S
W5Q9P7	Cofilin-1 (Fragment) OS=Ovis aries GN=CFL1 PE=4 SV=1 - [W5Q9P7_SHEEP]	CP	S
W5Q9U8	Uncharacterized protein OS=Ovis aries GN=SYN3 PE=4 SV=1 - [W5Q9U8_SHEEP]		S
W5QA68	Uncharacterized protein OS=Ovis aries GN=SORL1 PE=4 SV=1 - [W5QA68_SHEEP]	CP	S
W5QAV4	Piwi-like protein OS=Ovis aries GN=PIWIL3 PE=3 SV=1 - [W5QAV4_SHEEP]		S
W5QB61	Peptidyl-prolyl cis-trans isomerase (Fragment) OS=Ovis aries GN=FKBP1A PE=4 SV=1 - [W5QB61_SHEEP]	CP	S
W5QBL7	Uncharacterized protein OS=Ovis aries GN=ARPC1B PE=4 SV=1 - [W5QBL7_SHEEP]	CP	
W5QBT0	Uncharacterized protein (Fragment) OS=Ovis aries GN=RPP40 PE=4 SV=1 - [W5QBT0_SHEEP]	CP	
W5QBW5	Uncharacterized protein OS=Ovis aries GN=LBP PE=4 SV=1 - [W5QBW5_SHEEP]	CP	S
W5QD33	Uncharacterized protein OS=Ovis aries GN=PHLDB2 PE=4 SV=1 - [W5QD33_SHEEP]	CP	
W5QEH0	Uncharacterized protein (Fragment) OS=Ovis aries GN=TWF1 PE=4 SV=1 - [W5QEH0_SHEEP]		S
W5QF22	Uncharacterized protein OS=Ovis aries PE=3 SV=1 - [W5QF22_SHEEP]	CP	
W5QF31	Uncharacterized protein (Fragment) OS=Ovis aries GN=LOC101119845 PE=4 SV=1 - [W5QF31_SHEEP]	CP	
W5QF68	Uncharacterized protein OS=Ovis aries GN=FBXO48 PE=4 SV=1 - [W5QF68_SHEEP]	CP	
W5QFH0	Uncharacterized protein OS=Ovis aries GN=ARSA PE=4 SV=1 - [W5QFH0_SHEEP]	CP	
W5QFI4	Amino acid transporter OS=Ovis aries GN=SLC1A4 PE=3 SV=1 - [W5QFI4_SHEEP]	CP	
W5QG17	Adenylyl cyclase-associated protein OS=Ovis aries GN=CAP1 PE=3 SV=1 - [W5QG17_SHEEP]		S
W5QGK4	Uncharacterized protein (Fragment) OS=Ovis aries GN=LOC101107274 PE=3 SV=1 - [W5QGK4_SHEEP]	CP	
W5QH23	Uncharacterized protein OS=Ovis aries GN=MASP1 PE=3 SV=1 - [W5QH23_SHEEP]	CP	
W5QH45	Uncharacterized protein OS=Ovis aries GN=KNG1 PE=4 SV=1 - [W5QH45_SHEEP]	CP	S
W5QH54	Uncharacterized protein OS=Ovis aries GN=FETUB PE=4 SV=1 - [W5QH54_SHEEP]	CP	S
W5QH64	Synaptosomal-associated protein OS=Ovis aries GN=SNAP23 PE=3 SV=1 - [W5QH64_SHEEP]		S
W5QHB1	Receptor protein-tyrosine kinase OS=Ovis aries GN=EPHB3 PE=4 SV=1 - [W5QHB1_SHEEP]	CP	
W5QHD2	Uncharacterized protein OS=Ovis aries GN=RPL18 PE=4 SV=1 - [W5QHD2_SHEEP]	CP	
W5QHZ5	Uncharacterized protein OS=Ovis aries PE=4 SV=1 - [W5QHZ5_SHEEP]	CP	S
W5QI54	Uncharacterized protein (Fragment) OS=Ovis aries GN=FAM63B PE=4 SV=1 - [W5QI54_SHEEP]	CP	
